# Design and validation of fibroblast activation protein alpha targeted imaging and therapeutic agents

**DOI:** 10.7150/thno.41409

**Published:** 2020-04-27

**Authors:** Jyoti Roy, Suraj U Hettiarachchi, Miranda Kaake, Ramesh Mukkamala, Philip S Low

**Affiliations:** Department of Chemistry and Institute for Drug Discovery, Purdue University, West Lafayette, Indiana 47907, United States.

**Keywords:** Fibroblast activation protein alpha, cancer-associated fibroblast, tumor microenvironment, tubulysin therapy, chemotherapy, optical imaging, SPECT imaging

## Abstract

**Background**: Cancer-associated fibroblasts (CAFs) comprise a major cell type in the tumor microenvironment where they support tumor growth and survival by producing extracellular matrix, secreting immunosuppressive cytokines, releasing growth factors, and facilitating metastases. Because tumors with elevated CAFs are characterized by poorer prognosis, considerable effort is focused on developing methods to quantitate, suppress and/or eliminate CAFs. We exploit the elevated expression of fibroblast activation protein (FAP) on CAFs to target imaging and therapeutic agents selectively to these fibroblasts in solid tumors.

**Methods**: FAP-targeted optical imaging, radioimaging, and chemotherapeutic agents were synthesized by conjugating FAP ligand (FL) to either a fluorescent dye, technetium-99m, or tubulysin B hydrazide. In vitro and in vivo studies were performed to determine the specificity and selectivity of each conjugate for FAP in vitro and in vivo.

**Results**: FAP-targeted imaging and therapeutic conjugates showed high binding specificity and affinity in the low nanomolar range. Injection of FAP-targeted ^99m^Tc into tumor-bearing mice enabled facile detection of tumor xenografts with little off-target uptake. Optical imaging of malignant lesions was also readily achieved following intravenous injection of FAP-targeted near-infrared fluorescent dye. Finally, systemic administration of a tubulysin B conjugate of FL promoted complete eradication of solid tumors with no evidence of gross toxicity to the animals.

**Conclusion**: In view of the near absence of FAP on healthy cells, we conclude that targeting of FAP on cancer-associated fibroblasts can enable highly specific imaging and therapy of solid tumors.

## Introduction

In addition to malignant cells, solid tumors are commonly comprised of multiple stromal cell types including lymphocytes, tumor-associated macrophages, myeloid-derived suppressor cells, endothelial cells and fibroblasts. For the most part, these stromal cells support tumor growth and inhibit its immune-mediated eradication 1, 2. Like other stromal cells, cancer associated fibroblasts (CAFs) accumulate during tumor development and are subsequently reprogrammed by the tumor to release growth factors 3, angiogenesis-stimulating cytokines 4, immunosuppressive agents 5, and extracellular matrix proteins that rigidify the tumor mass and restrict entry of drugs and immune cells 5, 6. Because these components collectively enhance tumor growth, facilitate metastasis, compromise immune-mediated tumor rejection, and restrict drug access to solid tumors 7, increased CAF numbers have been found to constitute a negative prognostic marker for patient survival 8, 9.

CAFs differ from normal fibroblasts in their biological appearance, function, growth patterns, and biomarkers 4. One unique biomarker that is abundant on CAFs but absent from quiescent fibroblasts is fibroblast activation protein alpha (FAP) 10. FAP is a transmembrane serine protease that facilitates remodeling of the extracellular matrix and thereby modulates important functions such as cell invasion, cell motility, cell adhesion, and angiogenesis 11. Because these functions can be critical to tumor progression and since FAP is virtually absent from healthy cells, 12 FAP has emerged as a promising target for selective delivery of imaging and therapeutic agents to solid tumors. Indeed, many FAP-targeted drugs are now undergoing preclinical or clinical development for treatment of a variety of cancers 12, 13, including anti-FAP antibodies 14, 15, FAP-activated prodrugs 16-19, FAP-targeted radiotherapeutic agents 20-22, and FAP-directed cancer imaging agents 23-25.

In the current study, we report the design and synthesis of a novel high affinity small molecule ligand of FAP (FL) for use in targeting attached drugs to FAP-expressing fibroblasts. We describe the use of this ligand for development of pre-operative radio imaging (^99m^Tc) and intra-operative fluorescence imaging agents for detection and removal of solid tumors. We further exploit our targeting ligand to deliver tubulysin B hydrazide, a highly toxic microtubule inhibitor, selectively to FAP-containing solid tumors. Although the FAP-tubulysin B conjugate is solely targeted to fibroblasts, we finally demonstrate that the conjugate can completely halt tumor progression.

## Materials and Methods

### Materials

Benzotriazol-1-yl-oxytripyrrolidinophosphonium hexafluorophosphate (PyBop), 1-[Bis(dimethylamino)methylene]-1H-1,2,3-triazolo[4,5-b]pyridinium 3-oxide hexafluorophosphate (HATU), N,N-Dimethylmethanamide (DMF), N-ethyl-N-isopropylpropan-2-amine (DIPEA), isopropyl alcohol (IPA,) dichloromethane (DCM), trifluoroacetic acid (TFA), Trifluoroacetic anhydride (TFAA), 1,2- ethanedithiol, triisopropylsilane (TIPS), sodium bicarbonate (NaHCO_3_) and all other chemical reagents were purchased from Sigma-Aldrich. Cell culture reagents such as Roswell Park Memorial Institute medium 1640 (RPMI 1640), Dulbecco's Modified Eagle's Medium (DMEM) were purchased from GIBCO, whereas fetal bovine serum (FBS), 1% penicillin-streptomycin, and 2mM glutamine were purchased from Life Technologies. Sodium pertechnetate (^99m^Tc) was obtained from Cardinal Health. HC Matrigel was purchased from BD Biosciences. 7-Amino-Actinomycin D (7-AAD) staining solution was procured from Abcam. All the molecules were purified using RP-HPLC (Agilent, C18 10 μm; 19 mm × 250 mm).

### Syntheses

All the synthesis steps are summarized in Supplementary Information (Schemes S1-5).

***Synthesis of Compound 3:*** To synthesize compound 3, anhydrous DMF compound 2 (1 eq), HATU (1 eq) and anhydrous DIPEA (5 eq) were added to a solution of compound 1 and stirred under argon atmosphere for 6 h (Scheme S1). The crude product was purified by RP-HPLC [A=2 mM ammonium acetate buffer (pH 7.0), B= acetonitrile, solvent gradient 0% B to 80% B in 35 min], yielding compound 3 (70-80%). LRMS-LC/MS (m/z): [M+H]^+^ calcd for C_13_H_21_F_2_N_3_O_4_, 321.32; observed mass for Boc deprotected molecule 222 (Figure S1).

***Synthesis of Compound 4:*** To a solution of compound 3 in anhydrous DCM, anhydrous pyridine (1 eq) and TFAA (1 eq) were added, and the reaction mixture was allowed to stir at room temperature for 1 h (Scheme S1). Progress of the reaction was monitored using analytical LC/MS. The crude product was purified by RP-HPLC [A= 2 mM ammonium acetate buffer (pH 7.0), B= acetonitrile, solvent gradient 0% B to 80% B in 35 min], yielding compound 4 (75% yield). LRMS-LC/MS (m/z): [M+H]^+^ calcd for C_13_H_19_F_2_N_3_O_3_, 303.31; observed mass for Boc deprotected molecule [M-Boc+ACN+H], 245 (Figure S2).

***Synthesis of Compound 7:*** Compound 4 was dissolved in TFA and stirred at room temperature for 30 min (Scheme S1). Progress of the reaction was monitored using analytical LC/MS. After completion of the reaction, TFA was evaporated by rotary evaporation to yield compound 5. Compound 5 was dried under high vacuum and used without further purification. LRMS-LC/MS (m/z): [M+H]^+^ cald for C_8_H_11_F_2_N_3_O, 203.19; observed mass 204.1 (Figure S3). To a solution of compound 5, in anhydrous DMF, compound 6 (1 eq), HATU (1 eq) and anhydrous DIPEA (5 eq) were added, and the reaction mixture was allowed to stir under argon atmosphere for 6 h (Scheme S1). Progress of the reaction was monitored by analytical LC/MS. The crude product was purified by RP-HPLC [A=2 mM ammonium acetate buffer (pH 7.0), B= acetonitrile, solvent gradient 0% B to 80% B in 35 min], yielding compound 7 (80%). LRMS-LC/MS (m/z): [M+H]^+^ calcd for C_20_H_25_F_2_N_5_O_4_, 437.45; observed mass for Boc deprotected molecule 338 (Figure S4).

***Synthesis of Compound 8 (FL):***Compound 7 was dissolved in TFA and stirred at room temperature for 30 min (Scheme S1). TFA was removed by rotary evaporation to yield compound 8. Compound 8 was dried under high vacuum and used without further purification. LRMS-LC/MS (m/z): [M+H]^+^ calcd for C_15_H_19_F_2_N_5_O_2_, 339.15; observed mass 339.1 (Figure S5).

***Synthesis of Compound 10:*** To a solution of compound 8 in anhydrous DMF, compound 9 (1 eq), HATU (1 eq), and anhydrous DIPEA (10 eq) were added and the reaction mixture was allowed to stir under argon atmosphere for 6 h (Scheme S2). Progress of the reaction was monitored by LC/MS. The crude product was purified by RP-HPLC [A=2 mM ammonium acetate buffer (pH 7.0), B= acetonitrile, solvent gradient 0% B to 80% B in 35 min] to yield compound 10 (80% yield). LRMS-LC/MS (m/z): [M+H]^+^ calcd for C_19_H_21_F_2_N_5_O_5_, 437.4; observed mass 438. ^1^H NMR (500 MHz, Deuterium Oxide) δ 8.58 - 8.47 (d, J = 4.8 Hz, 1H), 7.67 - 7.40 (m, 2H), 5.10 - 5.02 (dd, J = 9.1, 4.3 Hz, 1H), 4.64 - 4.54 (q, J = 7.2 Hz, 1H), 4.45 (s, 2H), 4.22 - 4.13 (m, 2H), 3.05 - 2.70 (m, 2H), 2.55 (s, 4H), 1.43 - 1.33 (d, J = 7.1 Hz, 3H) (Figure S9).

***Synthesis of FL-L1:*** Compound FL-L1 was prepared using Fmoc-protected solid phase peptide synthesis as described in Scheme S2. The final product was cleaved from the resin using the standard cocktail solution of TFA:water:TIPS: ethanedithiol (92.5%: 2.5%: 2.5%: 2.5%). Crude FL-L1 was purified by RP-HPLC [A=2 mM ammonium acetate buffer (pH 5.0), B= acetonitrile, solvent gradient 0% B to 80% B in 35 min] to yield (70%) FL-L1. LRMS-LC/MS (m/z): [M+H]^+^ calcd for C_22_H_26_F_6_N_6_O_6_S, 540.54; observed mass 541 (Figure S6).

***Synthesis of FL-L1-FITC and FL-L1-S0456:*** To synthesize the dye conjugates, purified FL-L1 and FITC maleimide (1 eq) or S0456 maleimide (1 eq) were dissolved in anhydrous DMSO containing anhydrous DIPEA (5 eq; Scheme S2). The reaction mixture was stirred under argon atmosphere at room temperature during which progress of the reaction was monitored by LC/MS. Following completion of the reaction after 1 h, the crude products (FL-L1-FITC and FL-L1-S0456) were purified by RP-HPLC [A=2 mM ammonium acetate buffer (pH 7.0), B= acetonitrile, solvent gradient 0% B to 80% B in 35 min]. The LCMS characterization of FL-L1-FITC (Yield 80%) and FL-L1-S0456 (Yield 80%) are LRMS-LC/MS (m/z): [M+H]^+^ calcd for C_46_H_39_F_2_N_7_O_13_S, 967.91; observed mass[M+H]^+^ 968.2, [M+H]/2^+^ 484.6 (Figure S7) and LRMS-LC/MS (m/z): [M+H]^+^ calcd for C_75_H_85_F_2_N_10_Na_3_O_22_S_5_, 1745.82; observed mass [M-2Na/2]+ 849.4 respectively (Figure S8).

***Synthesis of FL-L3:*** As described in Scheme S3, FL-L3 was synthesized by using Fmoc-protected solid phase peptide synthesis. All components of the conjugate were assembled on the H-Cys(Trt)2-chlorotrityl resin. The standard cocktail solution of TFA:water:TIPS:ethanedithiol (92.5%: 2.5%: 2.5%: 2.5%) was used to cleave the final conjugate from the resin. The crude product was purified by RP-HPLC [A=2 mM ammonium acetate buffer (pH 5.0), B= acetonitrile, solvent gradient 0% B to 80% B in 35 min] to yield (70%) the requisite product. LRMS-LC/MS (m/z): [M+H]^+^ calcd for C_37_H_52_F_2_N_10_O_11_S, 882.94; observed mass 882.0 (Figure S10).

***Formulation of non-radioactive FL-L3:*** Prior to radiolabeling with ^99m^Tc, FL-L3 was formulated according to a previously published procedure 26. Briefly, in argon purged water, 0.1 mg FL-L3, 80 mg sodium α-D-glucoheptonate, and 10 mg tin (II) hydrochloride were dissolved. The pH of the solution was adjusted to 6.8 ±0.2 with sodium hydroxide or hydrochloric acid and the final volume was adjusted to 10 mL using argon purged water. After transferring 1 mL to each of 10 vials, the above solutions were lyophilized, and the resulting powder was sealed in the vials under argon and stored at -20^o^C until further use.

***^99m^Tc labeling of FL-L3:***FL-L3 was radiolabeled according to a previously published procedure 26. Briefly, to a formulated vial of FL-L3, 1 mL of ^99m^Tc sodium pertechnetate (15 mCi) was added and the solution was heated for ~18 min at 100 ^o^C. After cooling to room temperature, the chelation efficiency of ^99m^Tc was confirmed by radio HPLC ( Water 600E Multisolvent Delivery System, Waters Nova-Pak C18 (3.9 × 150 mm) column, 1%-50% solvent B in 20 min (solvent A 0.1% TFA in water; solvent B acetonitrile) at a flow rate of 1 mL/min; Scheme S4) and the radiolabeled agent (>80 % yield) was used for in vitro and in vivo studies without any further purification.

***Synthesis of FL-L1-TubBH:*** FAP-targeted tubulysin B hydrazide was synthesized as described in Scheme S5. Briefly, FL-L1 was dissolved in argon purged HPLC grade water and adjusted to a pH 7.0 using a NaHCO_3_ saturated solution (in argon purged water). Disulfide activated tubulysin B hydrazide (1 eq) in THF was added to the reaction mixture and stirred at room temperature under an argon atmosphere. Progress of the reaction was monitored by analytical LRMS-LCMS. The crude product was purified by preparative RP-HPLC [A=2 mM ammonium acetate buffer (pH 7.0), B= acetonitrile, solvent gradient 0% B to 80% B in 35 min] to yield 95% of the desired product. LRMS-LC/MS (m/z): [M+H]^+^ calcd for C_67_H_93_F_2_N_13_O_17_S_3_, 1487.63; observed mass [M+2H]/2^+^ 744.04 (Figure S11).

### Cell culture

FaDu, HT29, MDA-MB231, KB, human FAP-transfected HLF(HLF-hFAP) cells were cultured in a medium consisting RPMI 1640, 10% FBS, 1% penicillin-streptomycin, 1% 2 mM glutamine at 37^o^C in a 5% CO_2_ and 95% humidified atmosphere. HLF cells were transfected with Kempbio FAP virus to induce expression of human FAP. HEK293-hFAP cells were created by transfecting parent HEK293 cells with a lentiviral vector (Cyagen Biosciences) containing the sequence for human FAP. After selection, using 2µL/mL of puromycin, FAP positive cells were cultured in DMEM medium supplemented with 2µL/mL of puromycin, 10% FBS, 1% penicillin-streptomycin, 1% 2 mM glutamine at 37^o^C in a 5% CO_2_ and 95% humidified atmosphere. Other cells used in this study was initiated by thawing frozen vials from a master stock saved from the original cell lines purchased from ATCC. All the experiments were performed within two to five passages following thawing of the cells. No mycoplasma test was performed for any of the cell lines.

### Flow Cytometry

HEK293-hFAP cells were incubated with either 7AAD or FL-L1-FITC+7AAD at room temperature. To confirm the specificity of FL-L1-FITC binding to HEK293-hFAP, at room temperature the cells were incubated with FL-L1-FITC+7AAD in the presence of 100-fold excess of FL. Unstained HEK293-hFAP cells were used as control. After 1 h cells in all the groups were washed 3x with the medium. Cell-bound fluorescence was determined by using BD flow cytometry and the data was analyzed using FlowJo. The experiment was done in duplicates.

### Confocal microscopy

FaDu (human head and neck cancer), HT29 (human colorectal cancer), MDA-MB231 (human triple-negative breast cancer), and KB (human papilloma) cancer cells (30,000) were plated on 4 well confocal plates and incubated with 100 mM FL-L1-FITC for 1 h at 37^o^C. The unbound fluorescence was removed by washing the cells 3x with medium, and cell-bound fluorescence was imaged using an Olympus confocal microscope. The experiment was done in triplicates.

### In vitro fluorescence binding assay

100,000 HEK293-hFAP cells were seeded in amine-coated 24 well plates, to ensure cell adherence. Upon formation of a monolayer, cells were incubated with various concentrations of the FL-L1-S0456 in the presence or absence of excess of FL. After incubation for 1 h, the cells were washed 3x with medium to remove to unbound fluorescence and dissolved in 1% SDS. The cell-bound fluorescence was measured using a fluorescence spectrophotometer set with a ʎ_ex_= 745 nm and ʎ_em_= 790 nm. Cell-bound fluorescence was plotted against various concentrations and the apparent K_d_ determined by using one-site binding (hyperbola) curve fit in GraphPad prism7. The experiment was done in triplicates.

### Animal husbandry

5-6 weeks old female athymic nu/nu mice were purchased from Harlan Laboratories and allowed access to normal rodent chow and water ad libitum. The animals were maintained on a standard 12 h light-dark cycle. All animal procedures were approved by the Purdue Animal Care and Use Committee.

### Ex vivo fluorescence imaging and biodistribution

Female nu/nu athymic (5-6 weeks old) were subcutaneously injected with 5 x 10^6^ FaDu, MDA-MB231, KB, or HT29 (with 20% matrigel) cells in 0.2 mL sterile PBS. Tumors were allowed to grow to approximately 200-500 mm^3^ before initiating imaging studies. Each tumor-bearing mouse was intravenously injected (via tail vein) with the 10 nmoles of fluorescent dye conjugate (FL-L1-S0456) either in the presence or absence of a 100-fold excess of unlabeled ligand (FL). Animals (n=5 for each group) were euthanized 2 h post-injection by CO_2_ asphyxiation and images were acquired using a Caliper IVIS Luminal II. After performing whole-body imaging, organs of interest were harvested and imaged to quantitate fluorescence accumulation. The image acquisition parameters were as follows: i) lamp level-high, ii) excitation-745 nm, iii) emission-ICG, iv) binning (M) 4M, (v) f-stop-4, (vi) FOV-12.5, (vii) acquisition time,5 s.

### Immunofluorescence staining

MDA-MB231 tumor-bearing mice (female nu/nu athymic) were euthanized 2 h post-injection of FL-L1-S0456 (10 nmoles, i.v). The excised tumors were embedded in OCT, frozen using chilled isopentane, and sectioned at 5 µm thickness using a cryostat. Sections were fixed in methanol for 5 min and rinsed using TRIS buffer with Tween 20 detergent (TBST). For immunofluorescence staining sections were incubated at room temperature with 2.5 % normal goat serum for 20 min, followed by incubation with primary antibodies [EpCAM (324228, Bio Legend); α SMA (ab21027, Abcam)] for 60 min. Tumor sections were rinsed twice with TBST and fluorescence-labeled secondary antibodies [Dylight 488 GoRb (DI1488, Vector); Alexa 555 DkoG (A21432, Invitrogen) were sequentially applied for 30 min. Sections were rinsed twice in TBST and incubated with DAPI (EN62248, Invitrogen) for 10 min. Slides were rinsed in water, and coverslipped using Prolong gold (P36934, Invitrogen). Stained sections were scanned, and images were obtained using Leica Versa scanner and ImageScope software.

### In vitro binding study using ^99m^Tc labeled FL-L3

HEK293-hFAP cells were seeded in amine coated 24 well plates and allowed to grow as a monolayer. Spent medium was replaced with medium containing various concentrations of ^99m^Tc labeled FAP conjugate (FL-L3). When competition with the radioligand was examined, cells were incubated with ^99m^Tc labeled FL-L3 in the presence of 100-fold excess of FL. After incubation for 2 h, cells were washed 3x with culture medium to remove unbound radioactive conjugate and dissolved in 0.5 mL of 0.25 N NaOH. Cell-bound radioactivity was counted using a gamma counter. The apparent K_d_ was determined by analyzing the data using Graph Pad Prism7 [one-site binding (hyperbola) curve fit]. The experiment was done in triplicates.

### Ex vivo radioactive imaging and biodistribtuion

Female SCID mice (5-6 weeks old) were subcutaneously implanted with MDA-MB231 cells (5 x 10^6^ cells per mouse). When tumors reached ~300 mm^3^, mice were intravenously injected with 150 µCi (5.5 MBq, 10 nmoles) ^99m^Tc labeled FL-L3 alone or in the presence of 100-fold excess of FL. After 2 h mice were sacrificed by CO_2_ asphyxiation, and imaging was performed using a KODAK Image Station. The parameters used for radio imaging were: acquisition time = 2 min, f-stop = 4, focal plane = 7, FOV = 200, binning = 4. For white light imaging, the parameters were: acquisition time = 0.05 s, f-stop = 11, focal plane = 7, FOV = 200, with no binning. For the biodistribution study, necropsy was performed to collect the organs/blood/tissues. Radioactivity associated with each of the organs/blood/tissues was determined by using a gamma counter.

### In vitro cytotoxicity studies

HLF-hFAP cells (10,000 cells) were incubated at room temperature for 1 h in 96 well plates containing various concentrations of FL-L1-TubBH. After incubation cell viability was determined using a “Cell Titer-Glo® Luminescent Cell Viability Assay kit” per the manufacturer's instructions. Luminescence signal associated with each well was determined and the IC_50_ was calculated using Dose-response-Inhibition curve [(Inhibitor) vs. normalized response] in GraphPad Prism 7. The experiment was done in triplicates.

### In vivo therapy study

5-6 weeks old female nu/nu athymic nude mice were subcutaneously injected with 5 x 10^6^ MDA-MB231 breast cancer cells. Tumors were measured in two perpendicular directions either daily or every other day during therapy, and their volumes were calculated as 0.5 x L x W^2^, where L is the longest axis (in millimeters), and W is the axis perpendicular to L (in millimeters). Once the tumor volume reached ∼100 mm^3^, mice were randomly divided into control, competition, or treatment groups (n=5 for each group) and therapy was initiated. Dosing solutions were prepared in sterile saline and injected intravenously via tail vein. Mice in the treatment arms were injected with 40 nmoles FL-L1-TubBH either daily or every other day, and mice in the competition group were co-injected with 100-fold excess of the FAP ligand (FL). Mice in control group were administered with 100 µL of sterile saline. To monitor therapeutic response tumor volumes were measured throughout the study period. Difference in tumor volumes were statistically analyzed (*student t-test*) on day 7 and day 17 after the treatment was initiated. As a measure of gross toxicity, mice were weighed prior to each administration of drug.

## Results

### Synthesis and design of a novel FAP ligand and its conjugates

**Figure 1:**
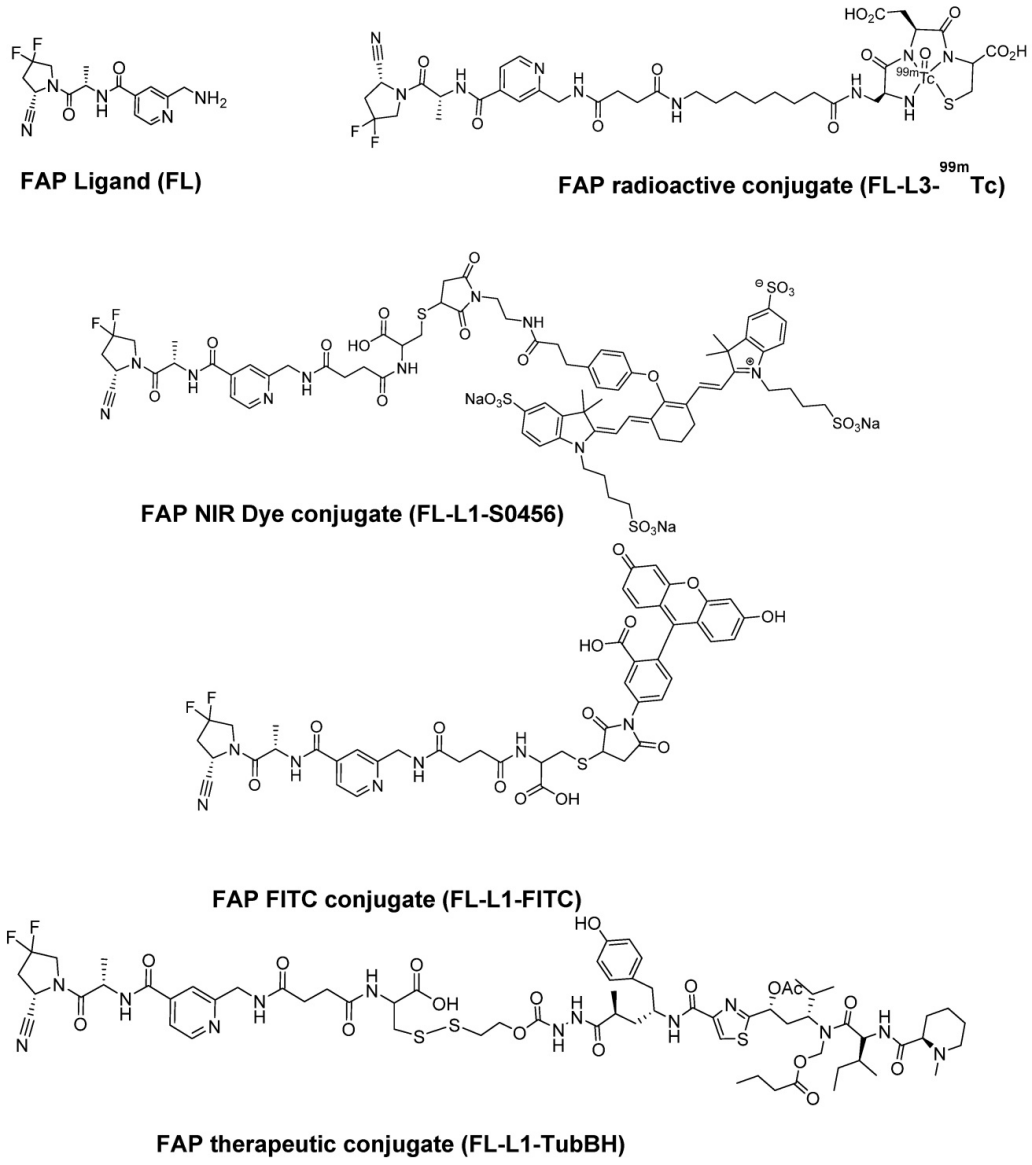
Chemical structure of FAP targeting ligand and its imaging and therapeutic conjugates. FAP ligand (FL), FAP NIR dye conjugate (FL-L1-S0456), FAP FITC conjugate (FL-L1-FITC), FAP radioactive technetium-99m conjugate (FL-L3-^99m^Tc), FAP tubulysin B hydrazide conjugate (FL-L1-TubBH).

Our fibroblast activation protein (FAP) ligand was designed by ligating those fragments of previous FAP ligands (FL) that were found by molecular docking studies to contribute most prominently to the specificity and affinity of FAP binding 27,28. As outlined in Scheme S2 and S3, the conjugates described below were assembled by linking FL to spacers of different length to generate either FL-L1 or FL-L3 via either solid or solution phase peptide chemistry. The FAP-targeted fluorescent dye conjugates (FL-L1-FITC and FL-L1-S0456; Figure 1) were then synthesized by coupling FL-L1 with either FITC maleimide or S0456 maleimide (Scheme S2). Due to the availability of 488 nm excitation on most confocal microscopes, the FITC conjugate was used primarily for confocal imaging of live FAP-expressing cells. In contrast, because of the deeper tissue penetration of NIR light, the S0456 dye conjugate was employed largely for optical imaging of tumor-bearing mice.

The FAP-targeted radioactive conjugate (FL-L3-^99m^Tc) was prepared by conjugating FL-L3 to a ^99m^Tc chelator via standard solid phase peptide synthesis (Scheme S3 and S4; Figure 1). FAP targeted fluorescent and radioactive conjugates were designed so that they do not release the imaging moieties whereas the therapeutic conjugate (FL-L1-TubBH) was assembled with a self-immolative cleavable linker to release the chemotherapeutic cargo. The intact FL-L1-TubBH conjugate was membrane impermeable and release of the tubulysin hydrazide was required to enable the drug to diffuse out of the endosome and into the cytoplasm where its microtubule was located (Scheme S5). In this latter case, self-immolation of the linker was designed to be triggered by the reducing environment in the endosomes 29.

### Affinity and specificity of FAP-targeted fluorescent conjugates

Expression of hFAP, on HEK293 cells, was determined by measuring cell-bound fluorescence associated with FL-L1-FITC (Figure S14). Blocking of FL-L1-FITC related fluorescence by unlabeled FAP ligand (FL), indicated FAP mediated uptake of FL-L1-FITC in HEK293-hFAP cells. Moreover, no FL-L1-FITC accumulation could be detected in non-transfected FAP negative cells (unpublished observation). Binding affinity of the FAP-targeted near-infrared fluorescent conjugate, FL-L1-S0456, for human FAP was determined by measuring the association of the FAP-targeted NIR dye with human FAP-transfected HEK293 cells. The resulting apparent K_d_ of 3.7 nM (Figure S12B) together with the observations that FL-L1-S0456 binding could be blocked by co-administration of excess of FAP ligand demonstrated that the interaction of FL-L1-S0456 with cell surface FAP occurred with high affinity and specificity.

**Figure 2:**
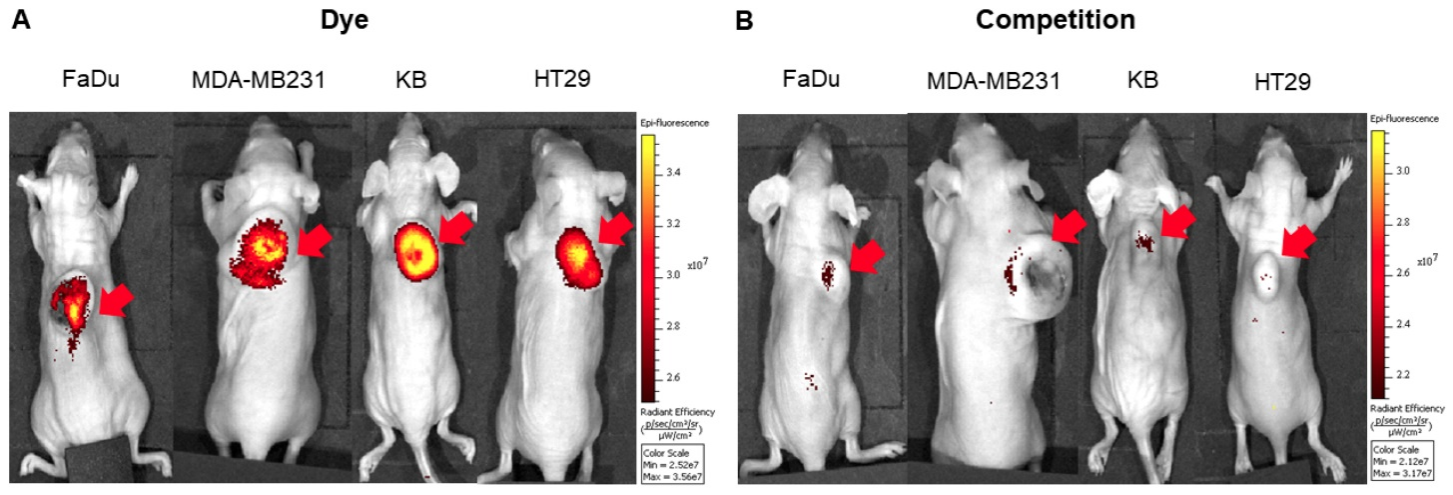
Representative in vivo whole-body optical imaging of FL-L1-S0456 in mouse xenografts. FaDu, MDA-MB231, KB, and HT29 tumor bearing mice were intravenously injected with FL-L1-S0456 (10 nmoles) either in the presence (B; competition) or absence (A; Dye) of excess of the FL. After 2 h post-injection mice were imaged using IVIVS Lumina. Images were acquired using the same imaging parameters across the tumor type and images in dye and competition groups were adjusted to same scale bar within each tumor type. Red arrow indicates tumor and n=5 for each group.

**Figure 3:**
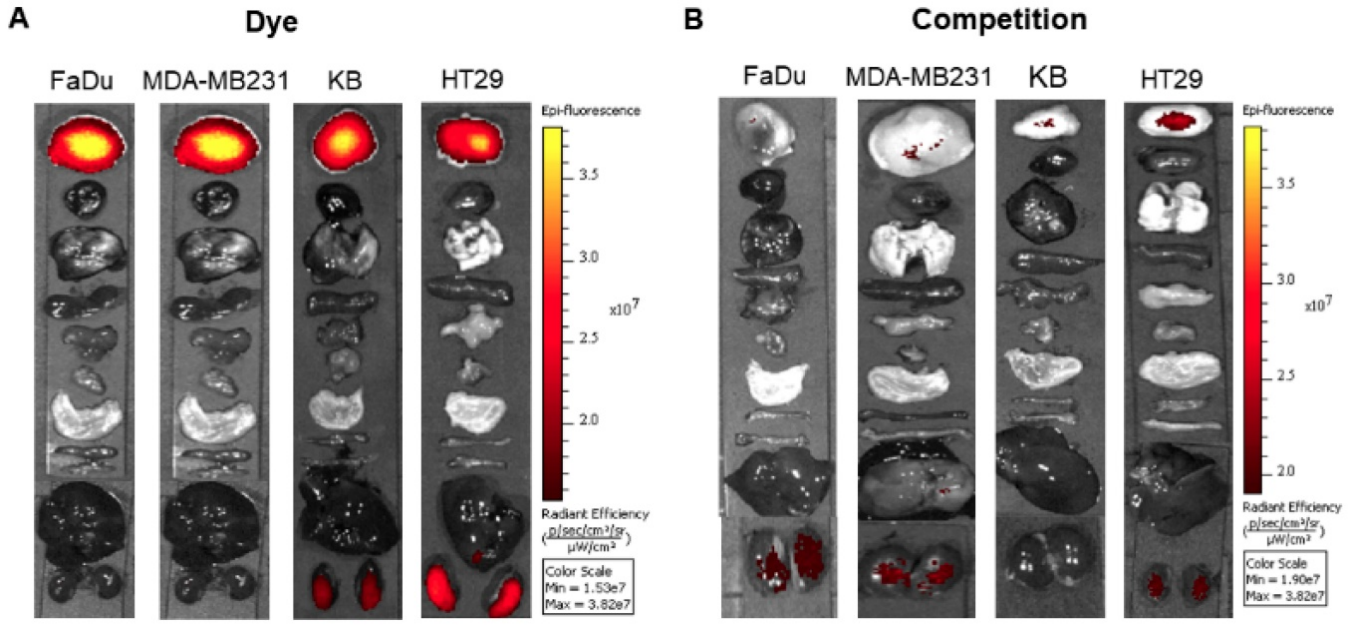
Representative in vivo biodistribution of FL-L1-S0456 in mouse xenografts. FaDu, MDA-MB231, KB, and HT29 bearing mice were intravenously injected with FL-L1-S0456 (10 nmoles) either in the presence (B; competition) or absence (A; Dye) of excess of the FL. Mice were euthanized 2 h post injection and biodistribution was performed. Organs and tissues were imaged using IVIVS Lumina. Images were acquired using the same imaging parameters across the tumor type and adjusted to same scale bar within each tumor type. List of organs (from top to bottom): Tumor, Heart, Lungs, Spleen, Pancreas, Muscle, Stomach, Small Intestine, Large Intestine, Liver, Kidney. N=5 for each group.

To explore the specificity of FL-L1-S0456 for CAFs in vivo, mice were implanted with four different cancer cell lines (i.e. FaDu, HT29, MDA-MB231, or KB tumors) that were shown to be FAP-negative by confocal microscopy (Figure S12A). Importantly, images of mice bearing all four tumor types taken 2 h after tail vein injection of FL-L1-S0456 demonstrated tumor-specific uptake of the dye (Figure 2A). That this uptake is mediated by CAFs is shown in Figure S12C, where the staining for EpCAM positive cancer cells is shown to be distinct from the staining for FAP positive CAFs. These data therefore suggest that uptake of the NIR dye conjugate is mediated primarily by FAP expressed on the cancer associated fibroblasts (CAFs). Moreover, since administration of excess of FAP targeting ligand (FL) was found to block the tumor uptake of FL-L1-S0456 (Figure 2B), it can be concluded that tumor accumulation of FL-L1-S0456 is FAP-mediated. Finally, to examine whether any other organs might also capture FL-L1-S0456, tissues from the above mice were resected and examined for fluorescence ex vivo. As shown in Figure 3A, other than the tumors, only kidneys showed uptake of the dye conjugate. Moreover, since the uptake of FL-L1-S0456 by the kidneys could not be competed by excess of FL, we concluded that this kidney fluorescence is not due to capture by a FAP-expressing cell, but rather due to elimination via renal excretion (Figure 3B).

### Affinity and specificity of the FAP-targeted radioactive conjugate

**Figure 4:**
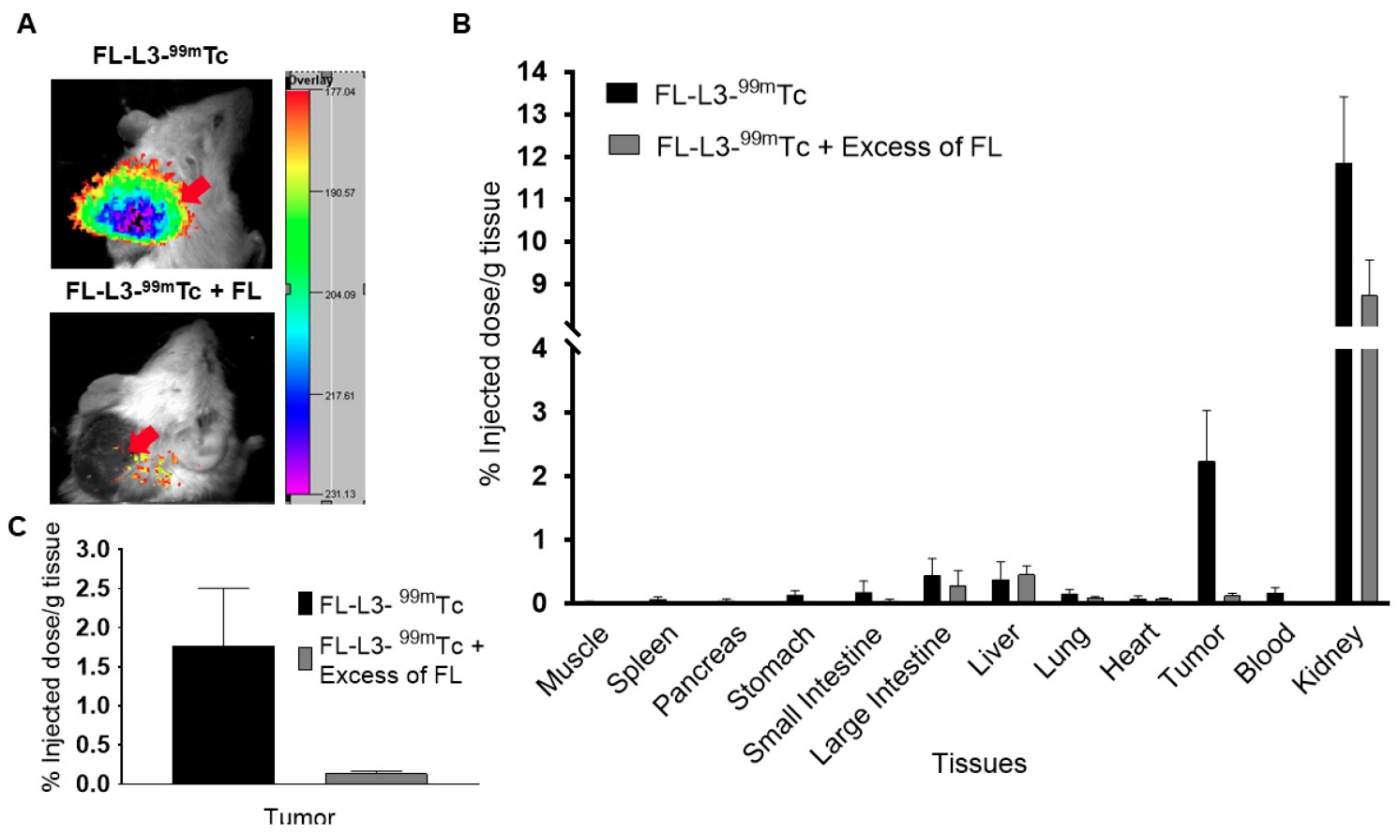
In vivo imaging and biodistribution of FAP-targeted technetium-99m conjugate (FL-L3-^99m^Tc). MDA-MB231 tumor xenografts were intravenously administered with 150 µCi (5.5 MBq) of FL-L3-^99m^Tc either in the presence (FL-L3-^99m^Tc +FL) or absence (FL-L3-^99m^Tc) of excess of FL. Two-hours post-injection, imaging (A) and biodistribution (B, C) was performed to determine radioactive content in various organs. Imaging was performed using Kodak imager. Tissue associated radioactivity was counted using a gamma counter. The error bar represents the mean %injected dose/g of tissue ± SD (n=5 for each group). Red arrow indicates the tumor.

As seen with the FAP-targeted NIR dye, the FAP-targeted radioactive conjugate (FL-L3-^99m^Tc) exhibited high affinity (K_d_= 10.5 nM; Figure S13) and specificity (i.e. blockade of uptake by excess FL) for FAP-transduced HEK293 cells in vitro. To establish a similar specificity for FAP-expressing cells in vivo, FL-L3-^99m^Tc was injected intravenously into mice implanted subcutaneously with FAP-negative MDA-MB231 cancer cells. As shown in Figures 4A, 4B, and 4C, FL-L3-^99m^Tc was observed to accumulate in MDA-MB231 solid tumors (2.23% ID/g) and a co-injection of excess of FL was seen to block this tumor uptake (0.13 ± 0.06 %ID/g), suggesting that tumor retention was FAP-mediated. Although radioactivity in the kidney was higher than in the tumor at this early time point (8.74 ± 1.44 %ID/g), it could not be blocked by co-administration of unlabeled FAP ligand suggesting that its presence in the kidneys is due to excretion from the body (Figure 4B). Because recruitment of CAFs into solid tumors can depend on tumor type, size, and infiltration of immune cells 30, 31, the lower accumulation of FL-L3-^99m^Tc in the MDA-MB231 tumors is likely a function of one of these variables.

### Effect of a FAP-targeted chemotherapeutic agent on tumor growth

**Figure 5:**
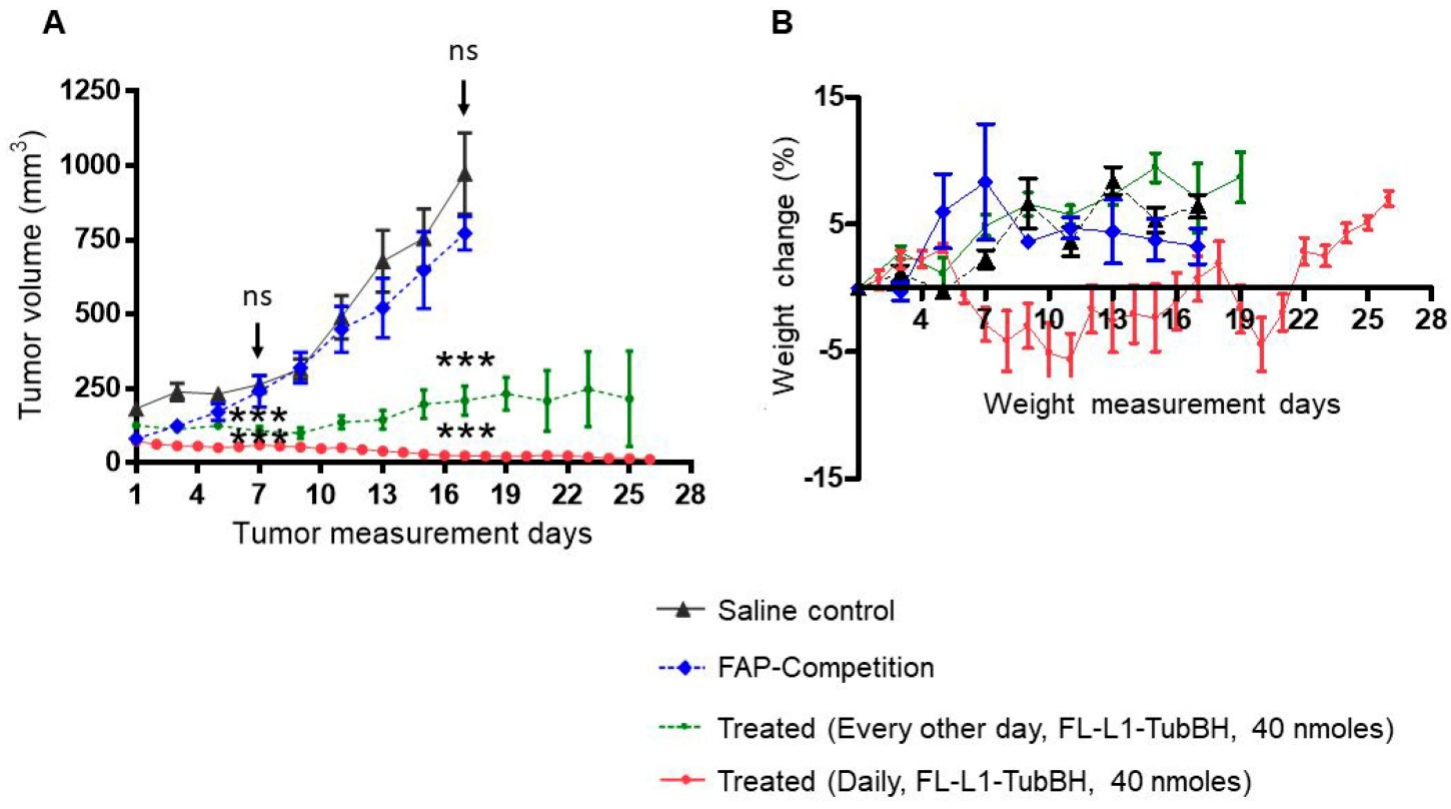
In vivo therapeutic efficacy of FL-L1-TubBH. MDA-MB231 tumor bearing mice were randomly divided into saline control, competition, and treated groups (n=5 for each group). Mice in saline control group were daily intravenously administered with saline whereas the mice in competition (FAP-Competition) group were co-injected with FL-L1-TubBH (40 nmoles) and FL (100-fold excess). Mice in treated groups were either administered every other day or daily with 40 nmoles of FL-L1-TubBH. Tumor volume (A) and weight (B) were monitored in mice in all the groups during the therapy. Each point in (A) represents mean tumor volume (mm^3^) ± SD, whereas each point in (B) represents mean weight (g) ± SD, *** represents p< 0.001 at day 7 and 17, ns indicates non-significant.

To investigate the ability of a FAP-targeted conjugate of tubulysin B hydrazide (FL-L1-TubBH) to suppress tumor growth, MDA-MB231 tumor-bearing mice were randomized into untreated controls (administered with saline alone), mice treated with FL-L1-TubBH, and mice treated with FL-L1-TubBH in the presence of excess blocking ligand, FL (competition). As shown in Figure 5A, saline-treated mice showed continuous tumor enlargement throughout the study. In contrast, mice treated with FL-L1-TubBH every other day exhibited delayed tumor growth, and mice treated daily with the same dose of FL-L1-TubBH demonstrated a complete response (Figure 5A). Co-injection of excess of FL together with FL-L1-TubBH (competition) totally prevented the antitumor activity of the therapeutic conjugate, demonstrating that tumor killing was indeed FAP-mediated. Compared to saline control group, mice in treated groups showed statistically significant reduction in tumor volumes (P<0.001 day 7 and day 17). On the other hand, the differences between tumor volumes of the mice in saline control and competition group were not statistically different (day 7: P <0.366 and day 17:P<0.216).

Because non-targeted tubulysin B was too toxic to administer at therapeutic concentrations 32, it was important to investigate the systemic toxicity of the FAP-targeted tubulysin B hydrazide conjugate at doses that displayed anti-tumor activity. As a crude measure of this systemic toxicity, all mice were weighed throughout the course of the study. As shown in Figure 5B, mice in the saline, competition, and every other day treated groups did not experience any weight loss, suggesting that the toxicities of the respective treatments were minimal. In contrast, the mice treated daily with FL-L1-TubBH displayed an initial decrease of ~5% in body weight, but eventually returned to the body masses of the other groups. Thus, although some initial toxicity was likely caused by the daily dosing regimen, the rapid recovery to normal body weight suggests that in general the FAP-targeted tubulysin B conjugate was well tolerated. Finally, since FAP expression is likely limited to CAFs within the tumor micro-environment, the ability of the FL-L1-TubBH conjugate to completely eradicate the tumor emphasizes on the vital role CAFs play in tumor survival.

## Discussion

Although our lab has focused on development of cancer-specific targeting ligands, the cell surface receptors that we have targeted to date have all resided primarily on the malignant cells (e.g. folate receptor alpha 29, 33, prostate specific membrane antigen 34, 35, cholecystokinin 2 receptor 36, 37, carbonic anhydrase IX 38, 39, neurokinin 1 receptor 40, and luteinizing hormone releasing hormone receptor 41, 42). Our motivation for this strategy was that specific killing of the cancer cells was considered the only sure approach to achieve complete tumor eradication. However, with expanding evidence that tumor stromal cells may be essential to cancer cell survival, by supplying such critical components as growth factors, immunosuppressive cytokines, extracellular matrix structures, and vascular endothelial growth factors, etc. 3-6, it seemed prudent to explore whether targeting the tumor stroma might prove indirectly effective in controlling tumor growth and metastasis. While regulatory T cells, exhausted CD8^+^ lymphocytes, tumor-associated macrophages, myeloid-derived suppressor cells, and vascular endothelial cells might have constituted similarly promising stromal targets, we elected to initially pursue cancer associated fibroblasts, because they i) are present in virtually all solid tumors 3, 43, ii) are not constantly mutating like cancer cells 44, iii) secrete growth factors that aid tumor growth 3, iv) release immunosuppressive cytokines that suppress immune rejection of cancer cells 5, and v) secrete collagen and other extracellular matrix proteins that limit immune cell penetration into solid tumors and facilitate tumor cell metastasis to distant sites 45. And while much has been achieved in the design of fibroblast specific ligands, enormous opportunities remained to exploit this strategy for controlling cancer.

In this study, we have designed, synthesized, and characterized several FAP-targeted imaging and therapeutic agents both in vitro and in vivo. We first synthesized a FAP-targeted NIR dye that was exploited for imaging murine models of head and neck, breast, colorectal, and cervical cancers. Since the FAP-targeted NIR dye selectively accumulated in the stroma of all tested tumors, we believe that this agent has the potential to guide a surgeon in his/her effort to locate and resect all malignant lesions during a cancer surgery. Although conventional fluorescence-guided surgery agents deliver fluorescent dyes selectively to cancer cells 41, 38, 33, the nearly universal infiltration of CAFs into solid tumors suggests that specific imaging CAFs might contribute prominently to the intra-operative detection and resection of malignant tissue. Although two FAP-targeted fluorescent imaging agents have already been reported in the literature 53, 25, both agents rely on the catalytic activity of FAP (a serine proteinase) to release the fluorescent dye from a quencher without providing any mechanism to retain the released dye in the tumor.

Intravenously injected FL-L3-^99m^Tc was also found to locate in a tumor mass, allowing localization of malignant lesions in whole body images. Although a different FAP ligand linked to a PET imaging agent has already yielded excellent images of cancer patients in the clinic 46, no FAP-targeted ^99m^Tc imaging agent has been reported to date. As CAF-targeted therapies become more prominent in the clinic 18, 20, methods to image their efficacy will become increasingly important. Thus, a FAP-targeted ^99m^Tc or PET imaging agent could not only find application in solid tumor imaging, but also in monitoring a patient's response to CAF-directed therapies 46, 47.

Although chemotherapy constitutes the first line of treatment for many cancers, therapeutic agents are primarily designed to kill/modify cancer or immune cells within the tumor mass 48. Because CAFs can comprise up to 90% of tumor stromal cells 49 and since they contribute prominently to tumor growth and survival, it seemed prudent to explore whether our FAP ligand might be exploited to eradicate CAFs. Much to our surprise, delivery of tubulysin B to CAFs in the tumor microenvironment resulted in complete eradication of the tumor, presumably eliminating cancer and stromal cells alike. While this result was not anticipated, we can still offer two mechanisms to explain the data. First, capture, internalization, and release of tubulysin by the CAFs might allow the tubulysin B to diffuse into and kill adjacent cells within the same tumor mass. Tubulysin B is known to be highly toxic to virtually all cells 32 and if enters adjacent stromal or cancer cells, it would be expected to kill them. Second, it is also conceivable that destruction of CAFs might deprive proximal cancer cells of one or more components required for cancer cell survival. Loss of associated cancer cells might then promote dissemination of other stromal cells into other tissues of the body. Regardless of the mechanism, the data confirms a growing body of evidence that targeting the tumor stroma can contribute prominently to eradication of the tumor tissue 16, 17.

Finally, it has not escaped our notice that FAP-targeted drugs might find application in other diseases characterized by strong infiltration of activated fibroblasts such as organ fibrosis and myocardial infarctions 50-52. Thus, activated fibroblasts not only accumulate in cancer tissues, but they also concentrate in all fibrotic diseases and sites of tissue trauma 12. Whereas infiltration of fibroblasts is essential to the healing of traumatized tissue, accumulation of activated fibroblasts constitutes the cause of pathology in the fibrotic diseases since activated fibroblasts (myofibroblasts) secrete the collagen and extracellular matrix components that create the fibrosis 53. It would therefore seem logical that FAP targeting strategies might also be adapted for improved imaging and therapy of fibrotic diseases.

## Supplementary Material

Supplementary figures and schemes.

## Abbreviations

CAFs: cancer-associated fibroblasts
FAP: fibroblast activation protein alpha
FL: FAP ligand
SPECT: single-photon emission computerized tomography
NIR: near-infrared
TubBH: tubulysin B hydrazide
