# Supplementary material for: Design and validation of fibroblast activation protein alpha targeted imaging and therapeutic agents
Source: Theranostics. 2020 Apr 27;10(13):5778–89. doi: 10.7150/thno.41409 (PMC7254991; doi:10.7150/thno.41409)
Supplement: Supplementary file 1 — Supplementary figures and schemes. [file thnov10p5778s1.pdf]

**TABLE OF CONTENTS**

1. Scheme S1. Synthesis of FAP targeting ligand (FL).
2. Figure S1. Chemical structure and LC/MS trace of Compound 3.
3. Figure S2. Chemical structure and LC/MS trace of Compound 4.
4. Figure S3. Chemical structure and LC/MS trace of Compound 5.
5. Figure S4. Chemical structure and LC/MS trace of Compound 7.
6. Figure S5. Chemical structure and LC/MS trace of Compound 8 (FL).
7. Scheme S2. Synthesis of FL-L1-S0456 and FL-L1-FITC.
8. Figure S6. Chemical structure and LC/MS trace of FL-L1.
9. Figure S7. Chemical structure and LC/MS trace of FL-L1-FITC.
10. Figure S8. Chemical structure and LC/MS trace of FL-L1-S0456.
11. Scheme S3. Synthesis of FL-L3.
12. Figure S9. Chemical structure, LC/MS trace, and $^{1}$H NMR of Compound 10.
13. SFigure S10. Structure and LC/MS of FAP conjugate (FL-L3).
14. Scheme S4. Radioactive labeling of FL-L3 with $^{99m}$Tc and radio HPLC.
15. Scheme S5. Synthesis of FL-L1-TubBH.
16. Figure S11. Structure and LC/MS of FAP tubulysin B hydrazide conjugate (FL-L1-TubBH).
17. Figure S12. Confocal Imaging and in vitro binding affinity of FL-L1-FITC and FL-L1-S0456.
18. Figure S13. In vitro binding affinity and cytotoxicity plots.
19. Figure S14. Representative flow cytometry histograms to determine FAP expression on HEK293-hFAP cells and FAP mediated specific binding of FL-L1-FITC.

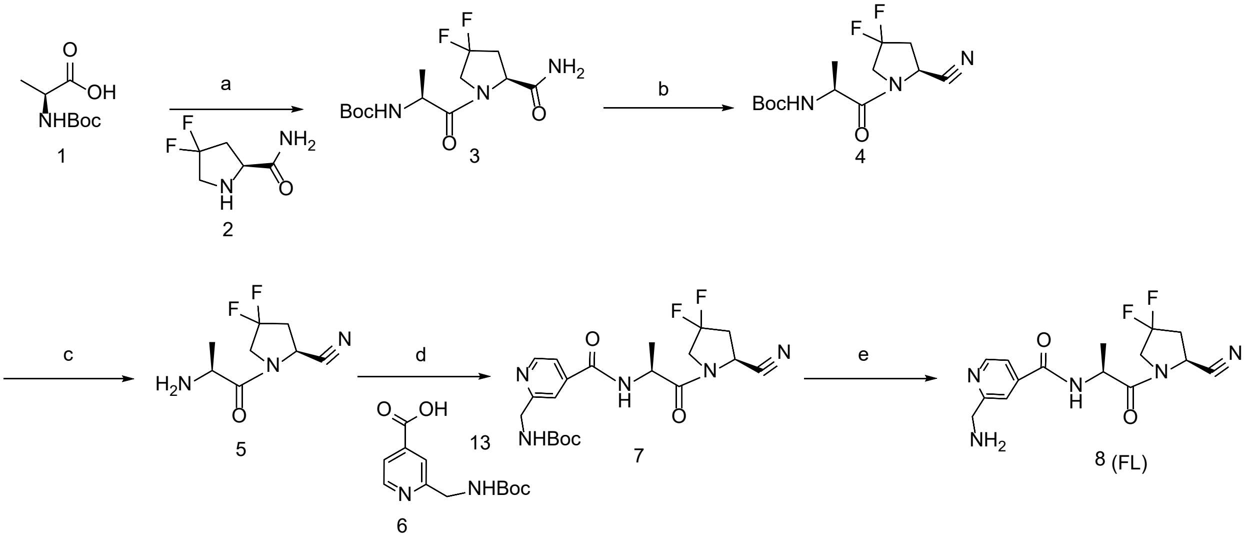


**Scheme S1.** Synthesis of FAP targeting ligand (FL). Reagents and conditions: a) HATU, Anhy. DIPEA, Anhy. DMF, rt; b) TFAA, Anhy. DCM, Anhy. Pyridine, rt; c) TFA, rt; d) HATU, Anhy. DIPEA, Anhy. DMF, rt; e) TFA, rt.

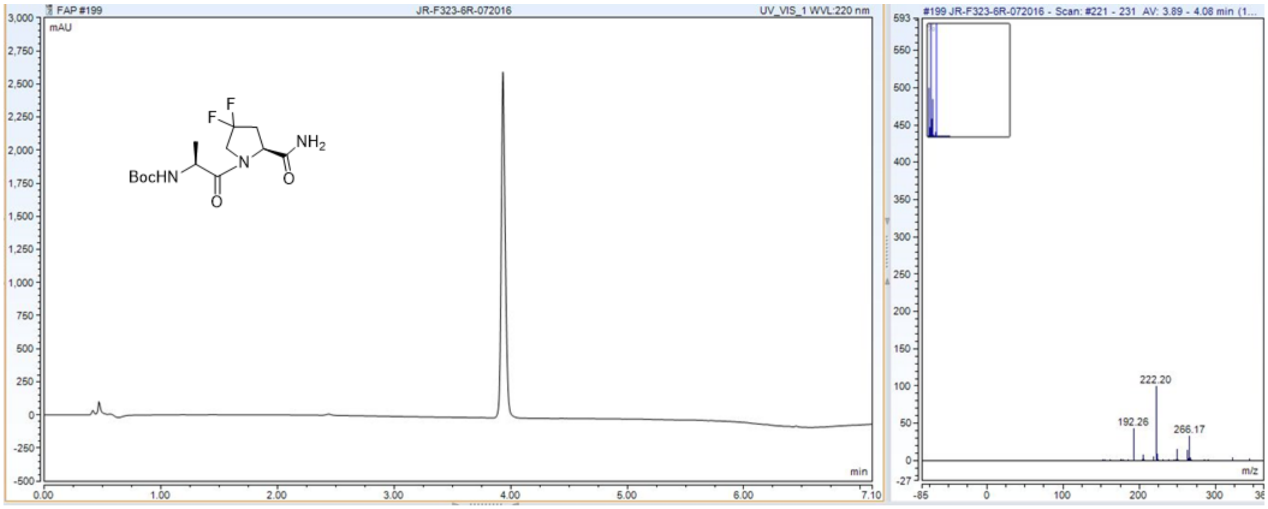


**Figure S1.** Chemical structure and LC/MS trace of Compound 3.

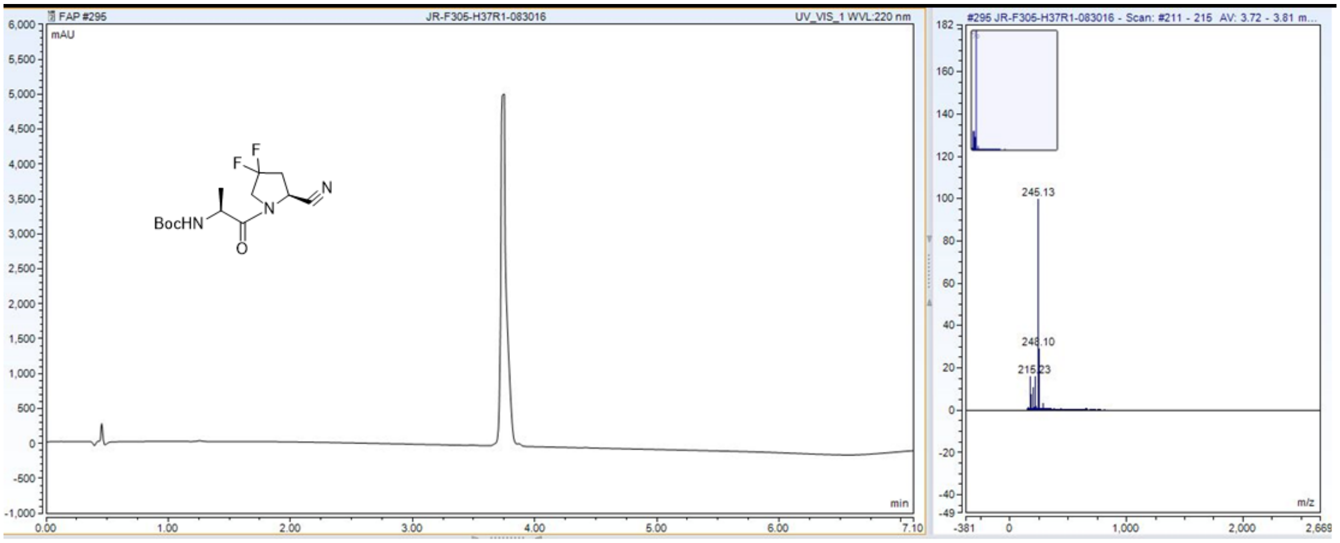


**Figure S2.** Chemical structure and LC/MS trace of Compound 4.

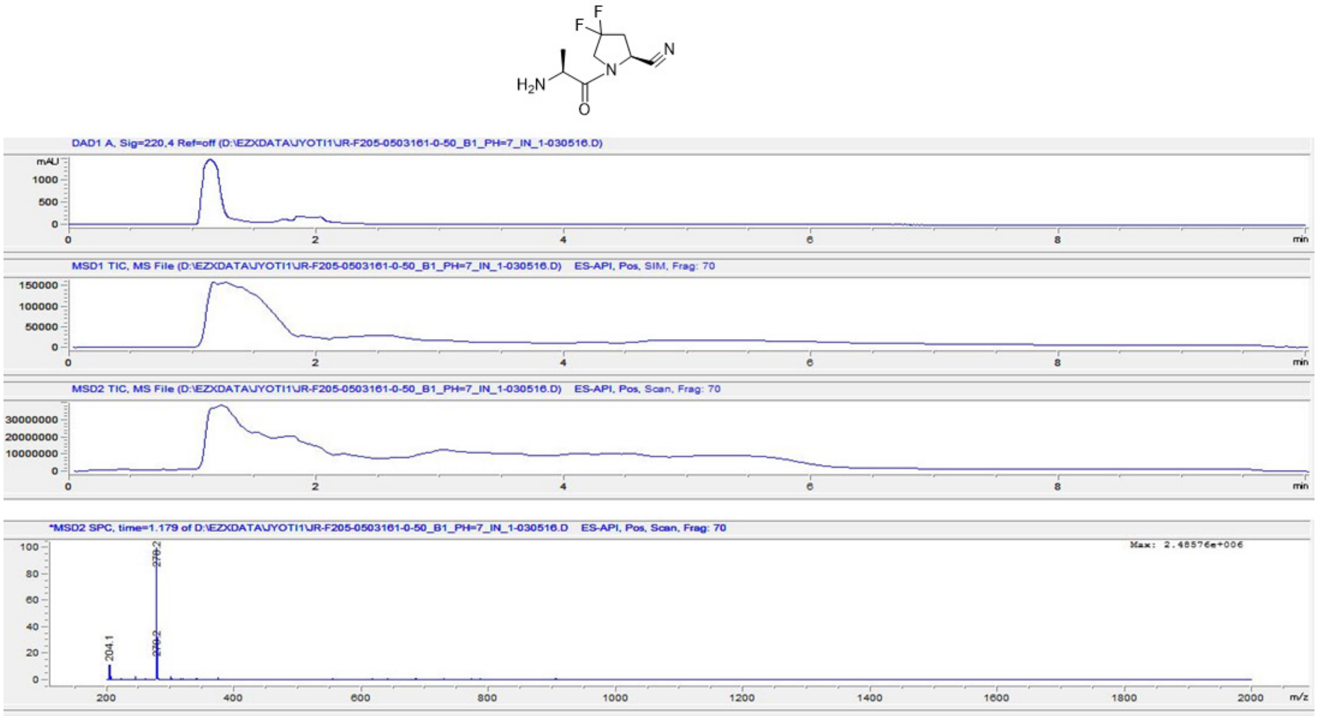


**Figure S3.** Chemical structure and LC/MS trace of Compound 5.

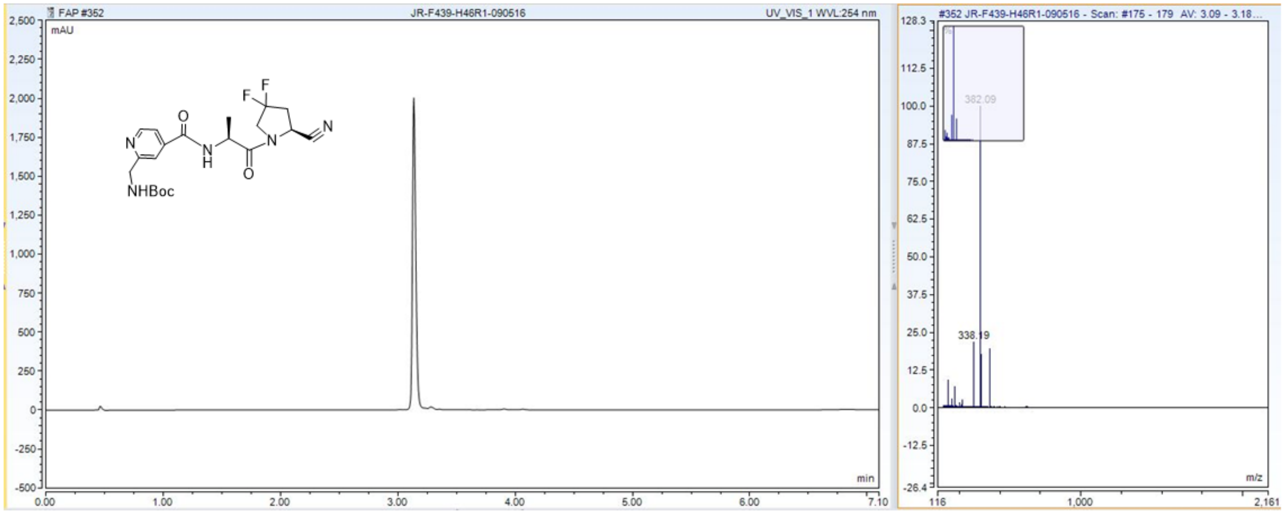


**Figure S4.** Chemical structure and LC/MS trace of Compound 7.

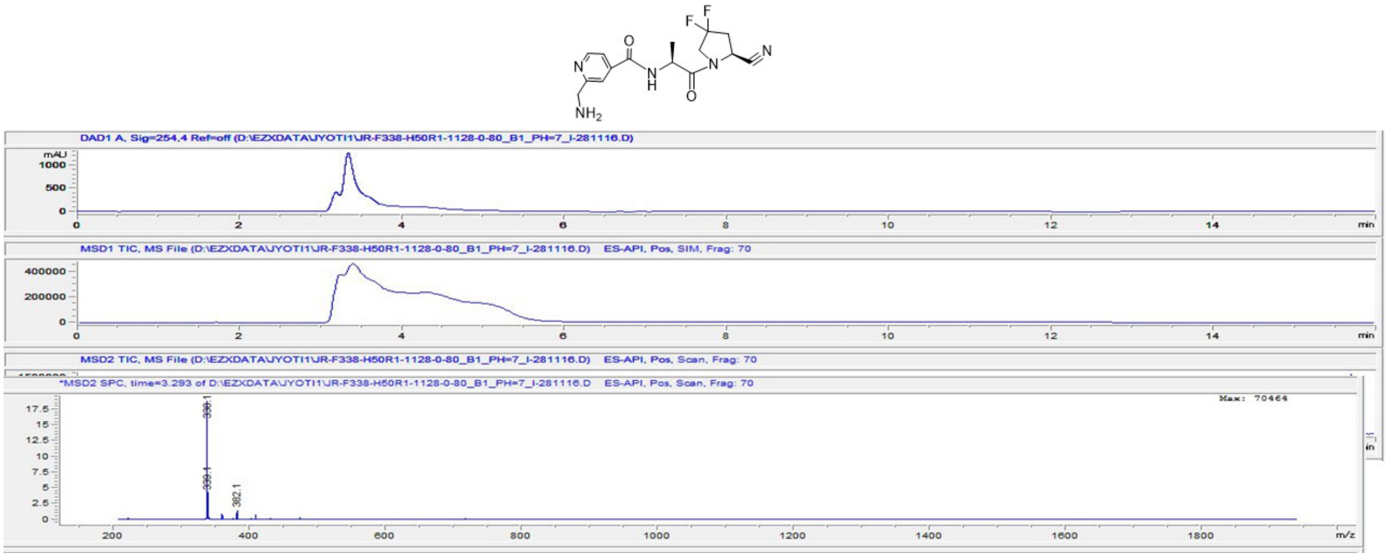


**Figure S5.** Chemical structure and LC/MS trace of Compound 8 (FL).

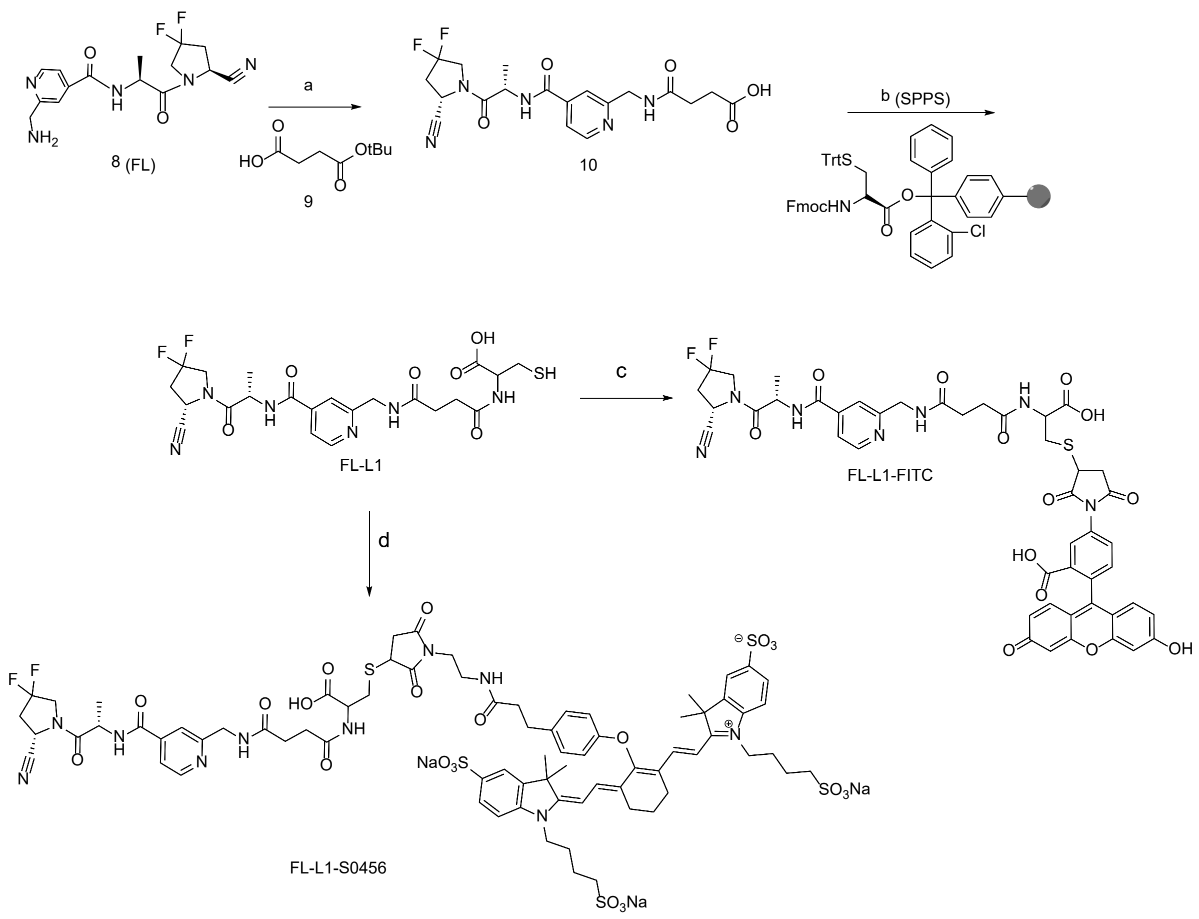


**Scheme S2.** Synthesis of FL-L1-S0456 and FL-L1-FITC. Reagents and conditions: a) (i) HATU (2.5 eq), Anhy. DIPEA (5 eq), Anhy. DMF, rt. (ii) TFA, rt b) PyBop (2.5 eq), Anhy. DMF, Any. DIPEA (5 eq), rt. (ii) TFA/$H_2O$/TIPS/EDT (92.5:2.5:2.5:2.5), 1 h. c) FITC maleimide (1 eq), DMSO, DIPEA (5 eq), rt. d) S0456 maleimide (1 eq), DMSO, DIPEA (5 eq), rt.

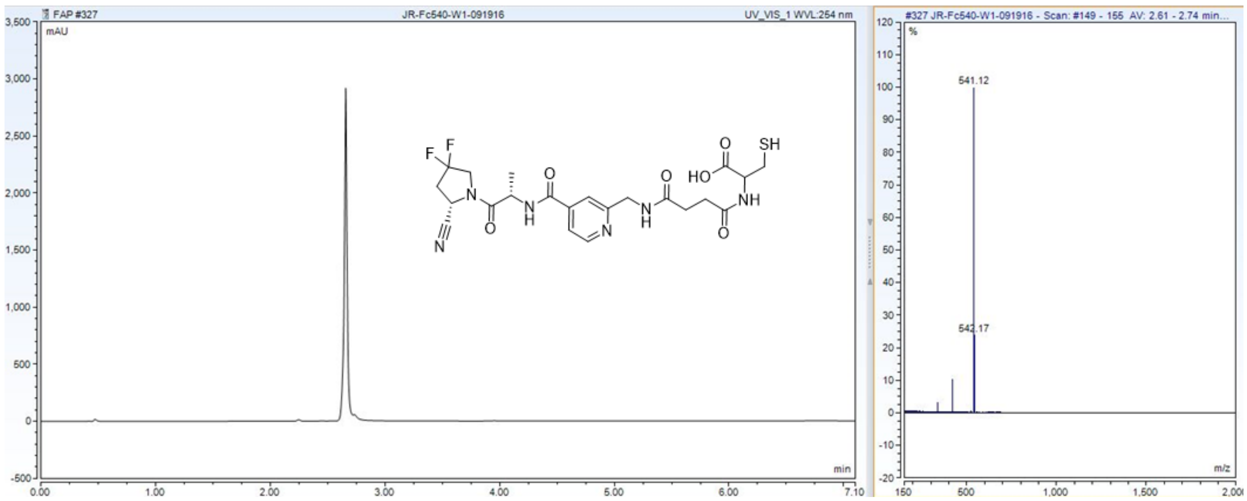


**Figure S6.** Chemical structure and LC/MS trace of FL-L1.

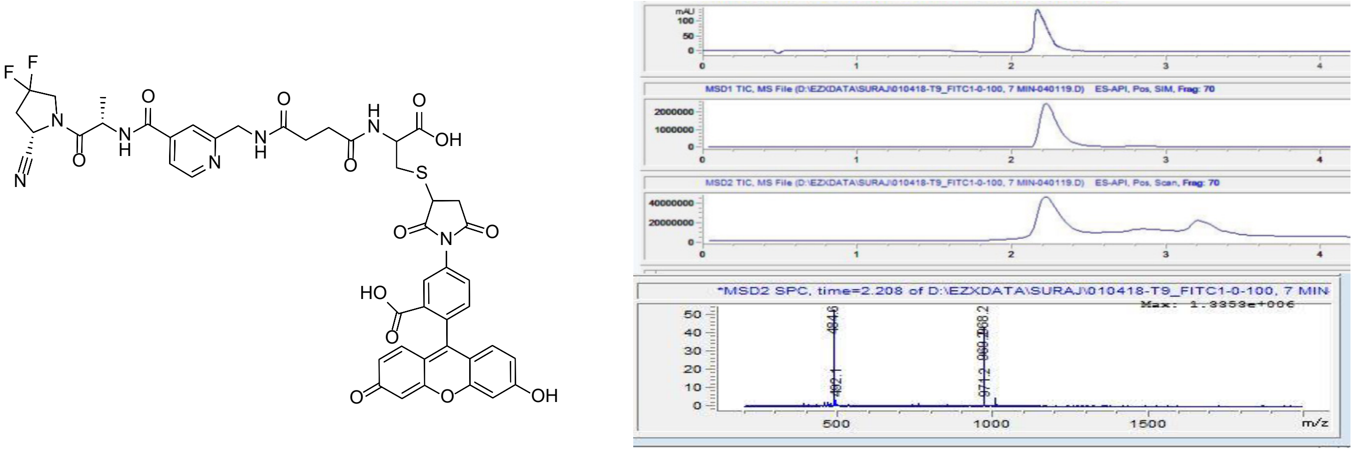


**Figure S7.** Chemical structure and LC/MS trace of FL-L1-FITC.

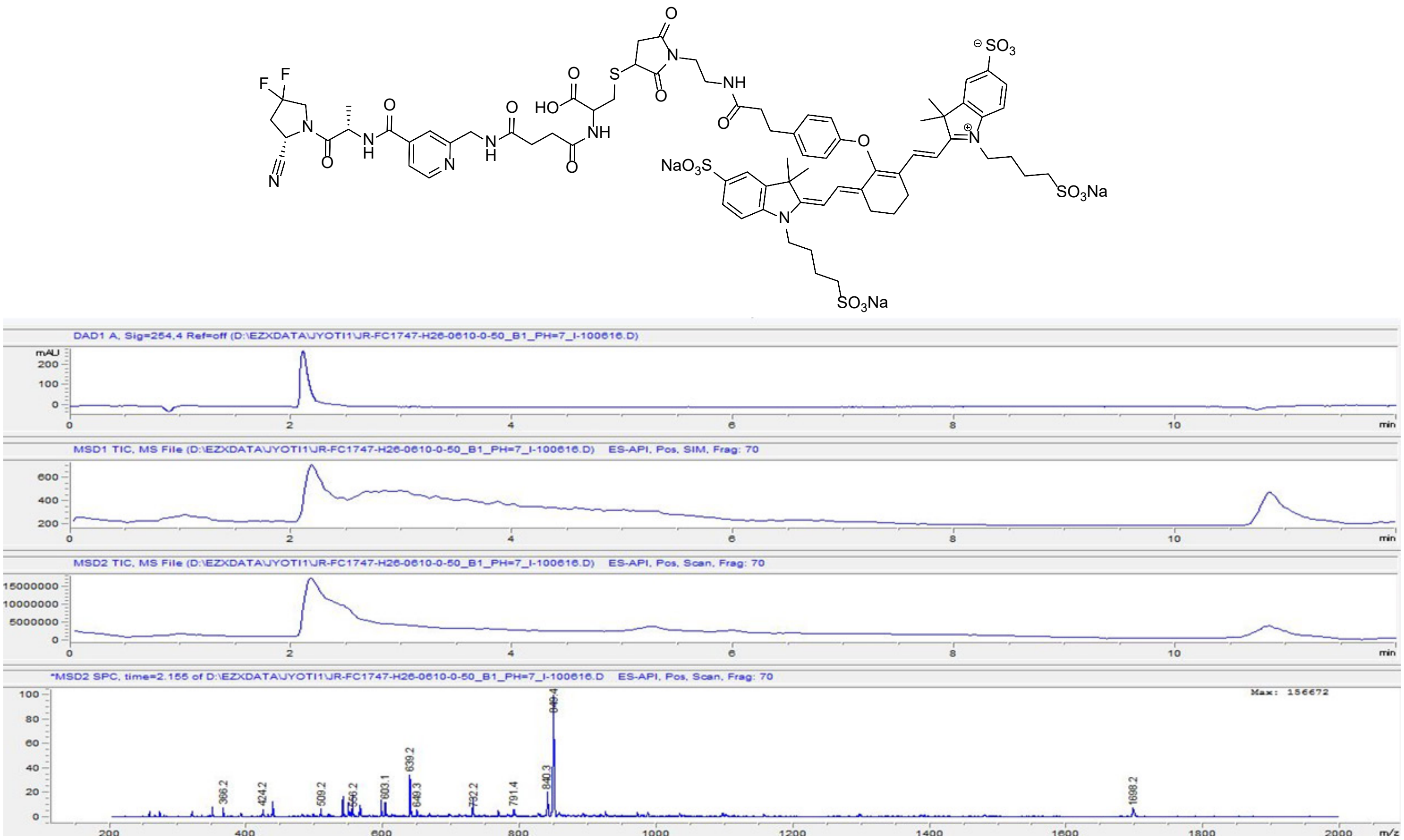


**Figure S8.** Chemical structure and LC/MS trace of FL-L1-S0456.

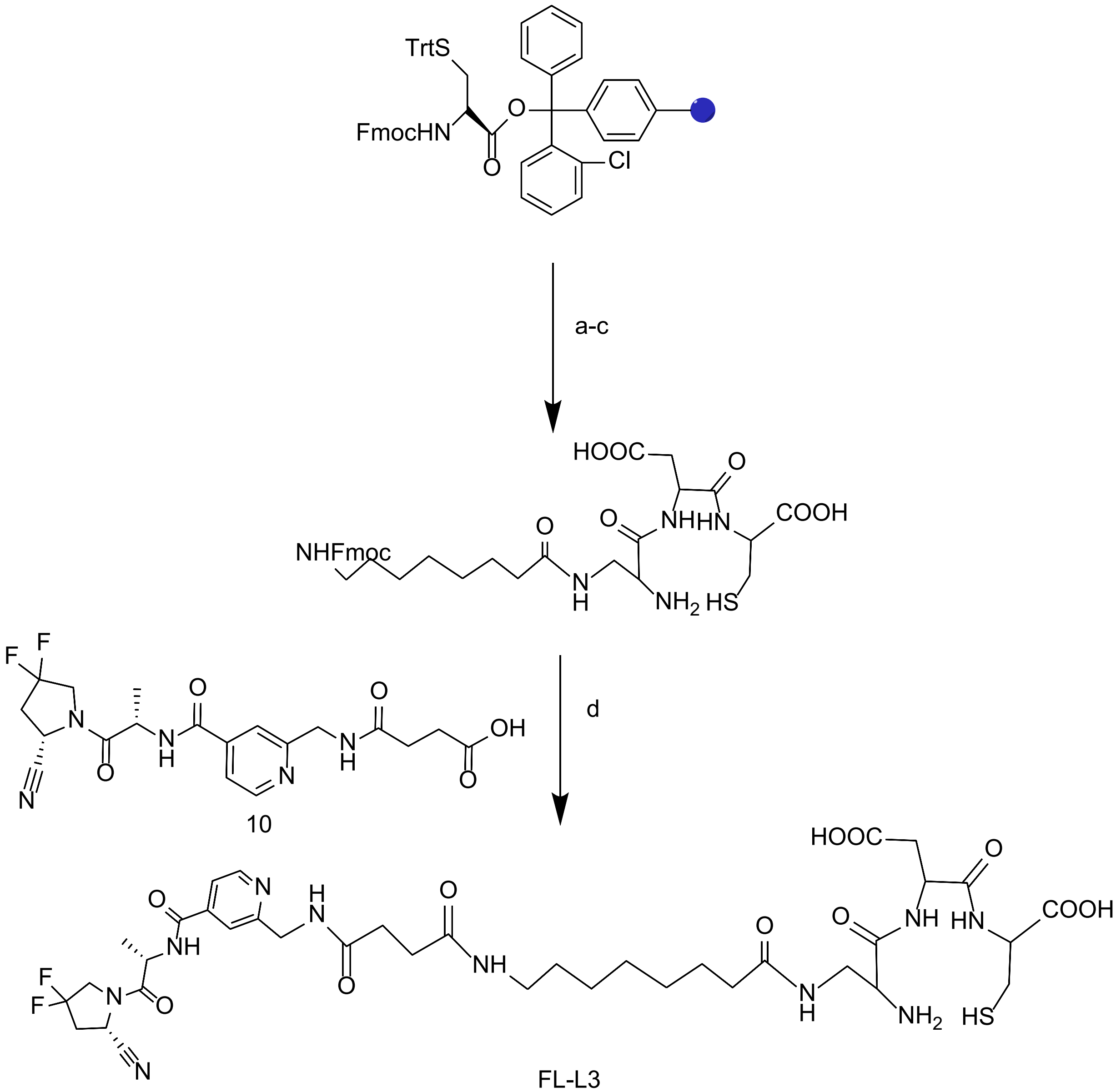


FL-L3

**Scheme S3.** Synthesis of FL-L3. Reagents and conditions: (a) (i) 20% piperidine/DMF, rt, (ii) Fmoc-Asp(OtBu), PyBop, DMF, DIPEA, (b) (i) 20% piperidine/DMF, rt,10 min (ii) Fmoc-diaminopropionic (DAP) acid, PyBop, DMF, DIPEA, (c) (i) 20% piperidine/DMF, rt, (ii) Fmoc-8-amino-octanoic acid, PyBop, DMF, DIPEA, (d) (i) 20% piperidine/DMF, rt,10 min (ii) Compound 10, PyBop, DMF, DIPEA, (iii) TFA/$H_2O$/TIPS/EDT (92.5:2.5:2.5:2.5), 1 h. The crude product was purified by using HPLC and characterized by using LC/MS.

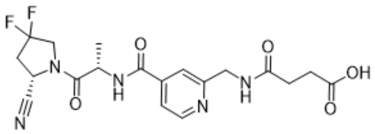


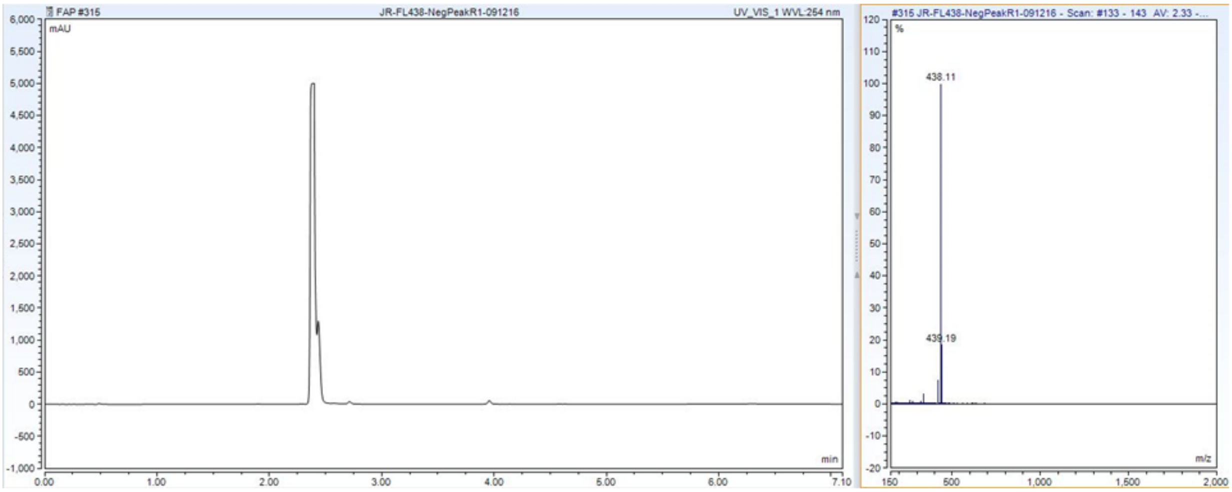


**Figure S9.** Chemical structure, LC/MS trace, and $^1$H NMR of Compound 10.

$^1$H NMR (500 MHz, Deuterium Oxide) δ 8.58 – 8.47 (d, J = 4.8 Hz, 1H), 7.67 – 7.40 (m, 2H), 5.10 – 5.02 (dd, J = 9.1, 4.3 Hz, 1H), 4.64 – 4.54 (q, J = 7.2 Hz, 1H), 4.45 (s, 2H), 4.22 - 4.13 (m, 2H), 3.05 – 2.70 (m, 2H), 2.55 (s, 4H), 1.43 – 1.33 (d, J = 7.1 Hz, 3H).

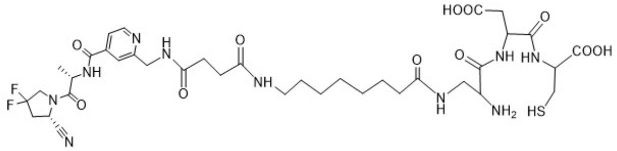


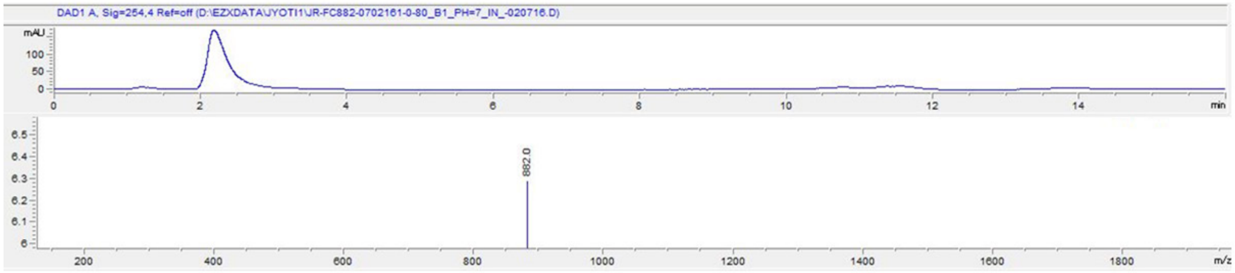


**Figure S10.** Structure and LC/MS of FAP conjugate (FL-L3).

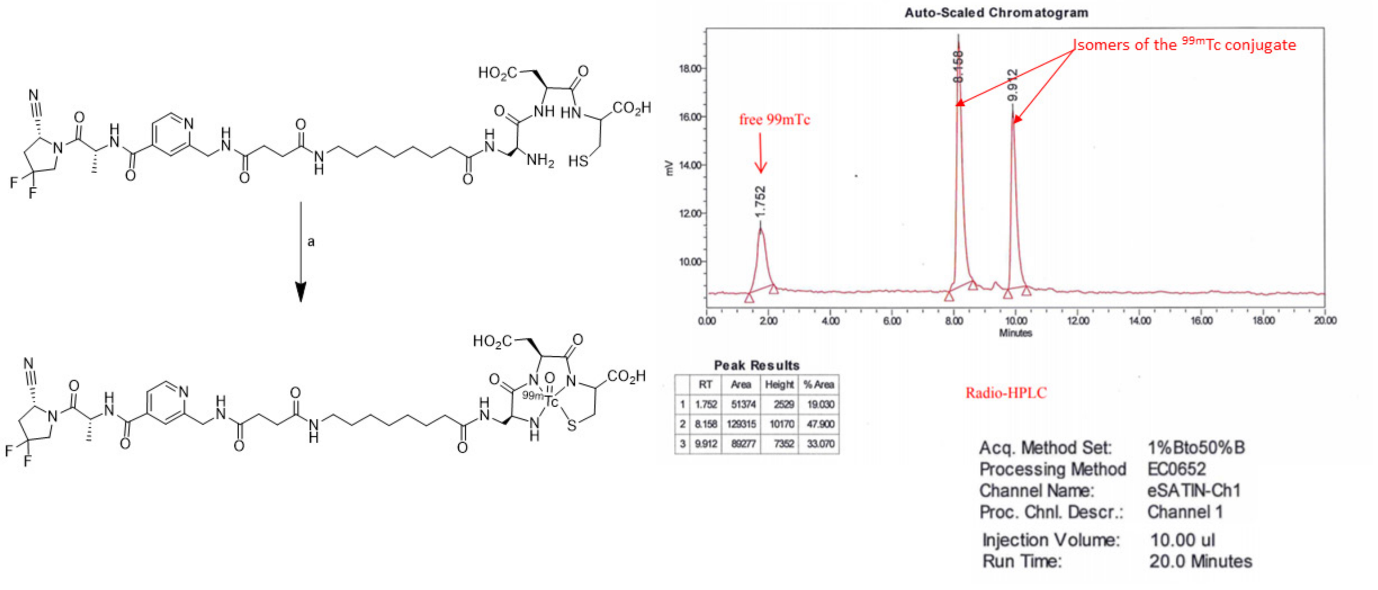


**Scheme S4.** Radioactive labeling of FL-L3 with $^{99m}$Tc and radio HPLC. Reagents and conditions: (a) $^{99m}$Tc sodium pertechnetate (15 mCi, 1 ml), 100 $^{o}$C, 18 min. Chelation efficiency was confirmed by radio HPLC.

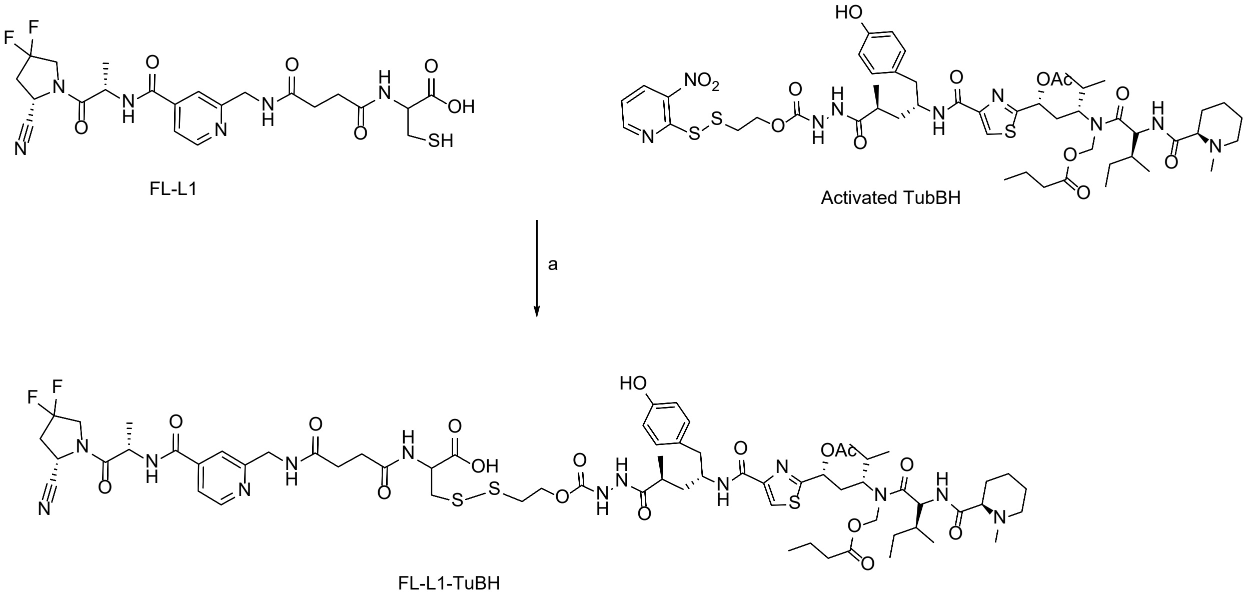


**Scheme S5.** Synthesis of FL-L1-TubBH. Reagents and conditions: a. (i) FL-L1, $H_2O/NaHCO_3$ (pH= 7.0-7.2), Argon, r.t. (ii) activated TubBH, anhydrous THF, argon, r.t.

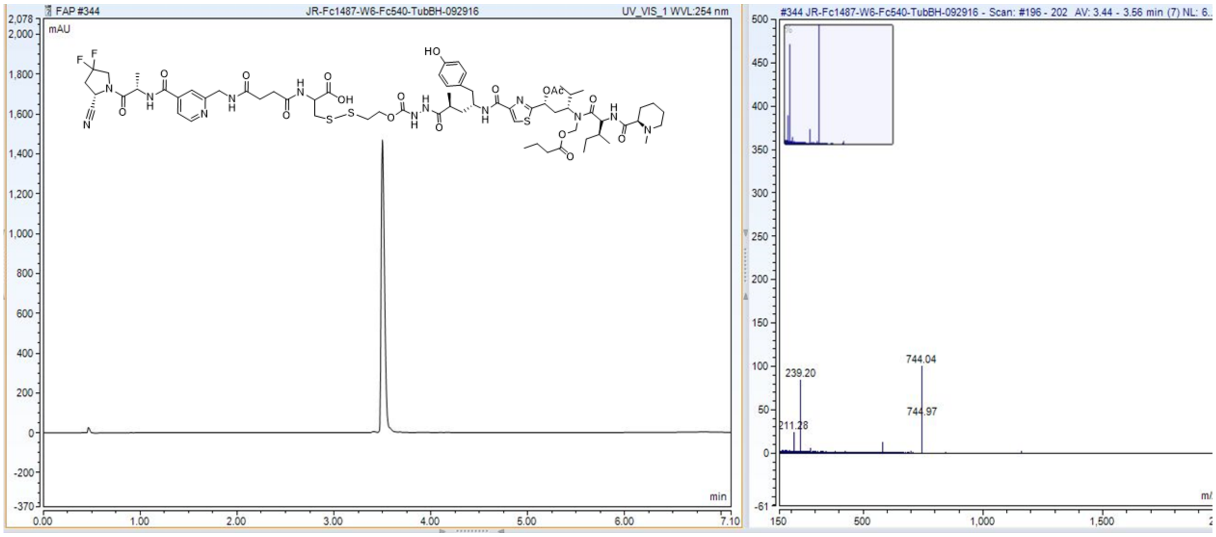


**Figure S11.** Structure and LC/MS of FAP tubulysin B hydrazide conjugate (FL-L1-TubBH).

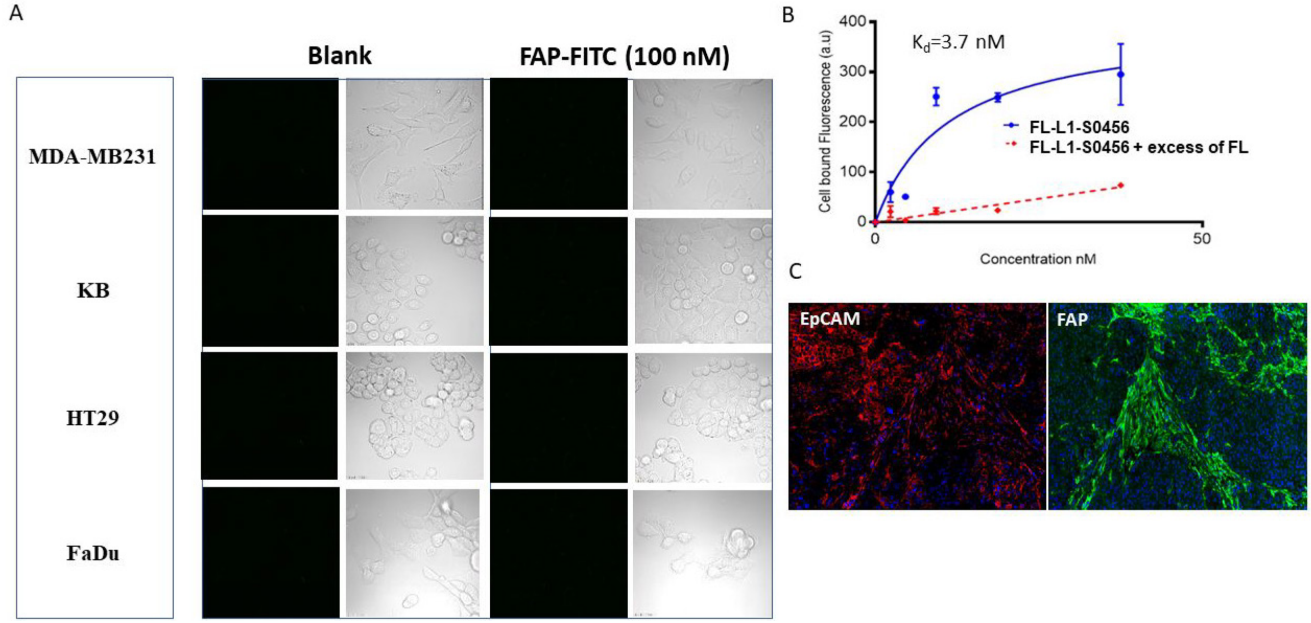


**Figure S12.** Confocal Imaging and in vitro binding affinity of FL-L1-FITC and FL-L1-S0456. A. The cancer cells were incubated with FL-L1-FITC (100 nM) at 37$^{o}$C. After incubating for 1 h the cells were washed with medium to remove unbound dye conjugate and observed under confocal microscopy. B. HEK293-FAP cancer cells were incubated with FL-L1-S0456 in the presence or absence of 100-fold excess of FL at 37$^{o}$C. After incubating for 1 h the cells were washed with medium to remove unbound dye conjugate. Cells were dissolved with 1% SDS and the cell bound fluorescence was measured using a fluorimeter. The $K_d$ represents the specific binding of FL-L1-S0456 for FAP. C. MDA-MB231

tumor- bearing mice were injected with FL-L1-S0456 (green), the tumors were excised, sectioned and stained for cancer epithelial cell marker (EpCAM, red) and nuclei (DAPI, blue).

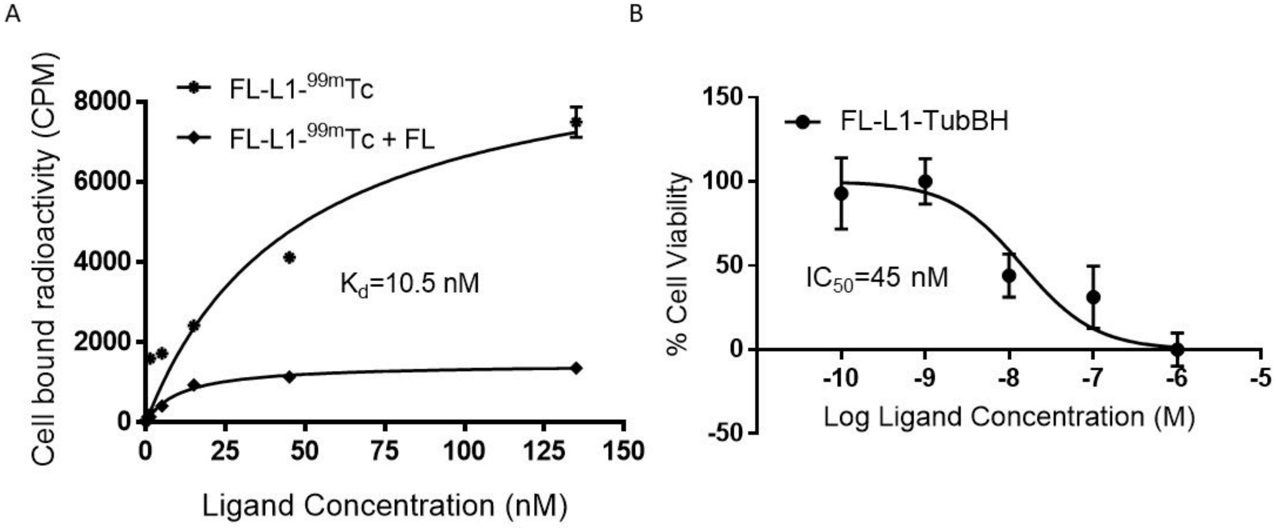


**Figure S13.** In vitro binding affinity and cytotoxicity plots. A. In vitro binding of FL-L1-$^{99m}$Tc. hFAP transfected HEK293 cells were incubated with various concentrations of FL-L3-$^{99m}$Tc either in the presence or absence of 100-fold excess of FL. The cell bound radioactivity was determined by gamma counting. $K_d$ value was determined by using GraphPad Prism 7. B. In vitro cytotoxicity of FL-L1-TubBH. HLF1 cells transfected with hFAP were incubated with various concentrations of FL-L1-TubBH and the cell viability was determined by using "*Cell Titer-Glo® Luminescent Cell Viability Assay kit".* The cell luminescence was determined and plotted in GraphPad Prism 7 to determine the $IC_{50}$.

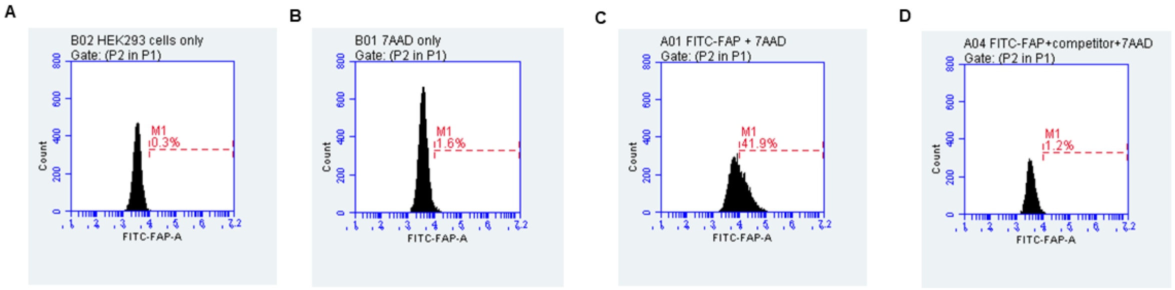


**Figure S14.** Representative flow cytometry histograms to determine FAP expression on HEK293-hFAP cells and FAP mediated specific binding of FL-L1-FITC. Histograms represent A) unstained control cells B) viable cells determined by 7AAD staining C) binding of cells to FL-L1-FITC dye conjugate (FITC-FAP+7AAD) D) binding of cells to FL-L1-FITC in the presence of 100-fold excess of FL to determine specificity (FITC-FAP+competition+7AAD).
